# Use of Patient-Reported Experience Measures in Pediatric Care: A Systematic Review

**DOI:** 10.3389/fped.2021.753536

**Published:** 2021-12-20

**Authors:** Sumedh Bele, Lorynn Teela, Muning Zhang, Sarah Rabi, Sadia Ahmed, Hedy Aline van Oers, Elizabeth Gibbons, Nicole Dunnewold, Lotte Haverman, Maria J. Santana

**Affiliations:** ^1^Department of Pediatrics, Cumming School of Medicine, University of Calgary, Calgary, AB, Canada; ^2^Department of Community Health Sciences, Cumming School of Medicine, University of Calgary, Calgary, AB, Canada; ^3^Patient Engagement Platform, Alberta Strategy for Patient-Oriented Research Support Unit, Calgary, AB, Canada; ^4^Psychosocial Department, Emma Children's Hospital Amsterdam UMC, University of Amsterdam, Amsterdam, Netherlands; ^5^Bachelor of Health Sciences Program, Cumming School of Medicine, University of Calgary, Calgary, AB, Canada; ^6^Bachelor of Sciences Program, Queen's University, Kingston, ON, Canada; ^7^Evidera, Oxford, United Kingdom; ^8^Health Sciences Library, University of Calgary, Calgary, AB, Canada

**Keywords:** patient-centered care, pediatrics, systematic review, routine clinical care, patient-reported experience measures (PREMs)

## Abstract

**Introduction:** Patient-reported Experience Measures (PREMs) are validated questionnaires, that gather patients' and families' views of their experience receiving care and are commonly used to measure the quality of care, with the goal to make care more patient and family-centered. PREMs are increasingly being adopted in pediatric population, however knowledge gaps exist around understanding the use of PREMs in pediatrics.

**Objective:** To identify and synthesize evidence on the use of PREMs in pediatric healthcare settings and their characteristics.

**Evidence Review:** Preferred Reporting Items for Systematic Reviews and Meta-Analysis guidelines governed the conduct and reporting of this review. An exhaustive search strategy was applied to MEDLINE, EMBASE, PsycINFO, Cochrane Library, and CINAHL databases to identify relevant peer-reviewed articles from high-income countries. Additionally, gray literature was searched to capture real-world implementation of PREMs. All the articles were screened independently by two reviewers in two steps. Data was extracted independently, synthesized, and tabulated. Findings from gray literature was synthesized and reported separately. Risk of bias for the studies identified through scientific databases was assessed independently by two reviewers using the National Institutes of Health Quality Assessment Tool for Observational Cohort and Cross-Sectional Studies.

**Results:** The initial search identified 15,457 articles. After removing duplicates, the title and abstracts of 11,543 articles were screened. Seven hundred ten articles were eligible for full-text review. Finally, 83 articles met the criteria and were included in the analyses.

Of the 83 includes studies conducted in 14 countries, 48 were conducted in USA, 25 in European countries and 10 in other countries. These 83 studies reported on the use of 39 different PREMs in pediatric healthcare settings. The gray literature retrieved 10 additional PREMs. The number of items in these PREMs ranged from 7 to 89. Twenty-three PREMs were designed to be completed by proxy, 10 by either pediatric patients or family caregivers, and 6 by pediatric patients themselves.

**Conclusion and Relevance:** This comprehensive review is the first to systematically search evidence around the use of PREMs in pediatrics. The findings of this review can guide health administrators and researchers to use appropriate PREMs to implement patient and family-centered care in pediatrics.

## Introduction

Pediatric healthcare systems around the world continue to evolve and are increasingly acknowledging the importance of delivering patient and family-centered care (PFCC) to improve all dimensions of quality, including patients' and families' experience with care received (1). Encouraged by the American Academy of Pediatrics, PFCC is key in the planning, delivery, and evaluation of healthcare that is grounded in mutually beneficial partnerships among healthcare providers, patients, and families. To improve and sustain the practice of PFCC, measuring patient and families' experience with the care received is necessary (2).

Patient-reported Experience Measures (PREMs) are validated questionnaires, that gather patients' and families' views of their experience receiving care. PREMs assess the impact of the process of care including communication between patient, their families and healthcare providers, information sharing, involvement of patients and their families in decision-making and are commonly used as indicators to evaluate the quality of care (2, 3). In the context of the Institute for Healthcare Improvement (IHI) Triple Aim Framework, the implementation of PREMs in healthcare leads to improved outcomes while lowering healthcare costs (4). In addition, it allows the voice of patients and their family to inform care improvement, an important concept included in the learning health system paradigm (5).

The growing adoption of PREMs in pediatric care requires the identification of appropriate PREMs and their subsequent use in healthcare settings. PREMs are centered around the experience while receiving care (e.g., hospital environment, ease of parking, call buttons near bed etc.) rather than clinical outcomes. Moreover, most of the validated PREMs are developed in high income countries which have comparable healthcare systems and services. Thus, the objective of this systematic review is to identify and synthesize evidence on the types of PREMs used in pediatric care, and their subsequent use in healthcare systems in high income countries to inform care improvement and support pediatric learning health systems paradigm.

## Methods

Preferred Reporting Items for Systematic Reviews and Meta-Analysis (PRISMA) guidelines governed the conduct and reporting of this review (6). The protocol has been registered with OSF (DOI 10.17605/OSF.IO/3RMNC).

MeSH (Medical Subject Headings) terms, keywords and their variations were used to develop a search strategy, which was initially applied to MEDLINE database to randomly screen 100 abstracts to refine this strategy. The final search strategy was applied to MEDLINE, EMBASE, PsycINFO, Cochrane Library, and CINAHL databases. Gray literature was searched through the websites of health institutes, pediatric hospitals, conferences, professional agencies, and search engines manually, which provided an overview of real-world implementation of PREMs.

Covidence was used for article screening and selection against pre-defined inclusion and exclusion criteria (Box 1) (8). In the first step, two independent reviewers screened titles and abstracts. Then, two reviewers independently screened selected articles by going through their full text. In both the steps, conflicts were resolved by discussion and consensus or by involving a third reviewer.

Box 1 **Inclusion and exclusion criteria.**

Inclusion Criteria

1. Population: Studies that focused only on pediatric populations (≤ 18 years).

2. Measure: Studies that implemented previously validated pediatric PREMs with explicit information regarding how the PREM was validated by mentioning either the validation process or referencing a previous article that described the validation and development process.

3. Geography: Included studies also needed to have been conducted in high-income countries, loosely defined by World Bank (7 ).

4. Articles published from January 2000 to April 2021.

Exclusion Criteria:

1. Population: Studies that focused on adult or general populations alongside pediatric populations.

2. Measure: Studies that utilized a non-validated PREM or a satisfaction survey. Editing a validated PREM threatens its validity, therefore we excluded studies where PREMs were either edited or researcher created their own questionnaires without conducting any validity testing.

3. PREM validation studies.

4. Study design: opinion pieces and reviews.

5. Language: Studies in languages other than English, French, Spanish, or Dutch.

Following screening, two reviewers independently extracted the data. Due to heterogeneity among the studies in both statistical and methodological domains, conducting a meta-analysis was neither warranted nor plausible. We instead synthesized the results inductively by tabulating identified PREMs in various geographic locations, their type, use and characteristics. Similarly, findings from gray literature are synthesized and reported separately.

Risk of bias for all the studies identified through scientific databases was assessed independently by two reviewers using the National Institutes of Health Quality Assessment Tool for Observational Cohort and Cross-Sectional Studies (9).

## Results

### Search Results

**Figure 1:**
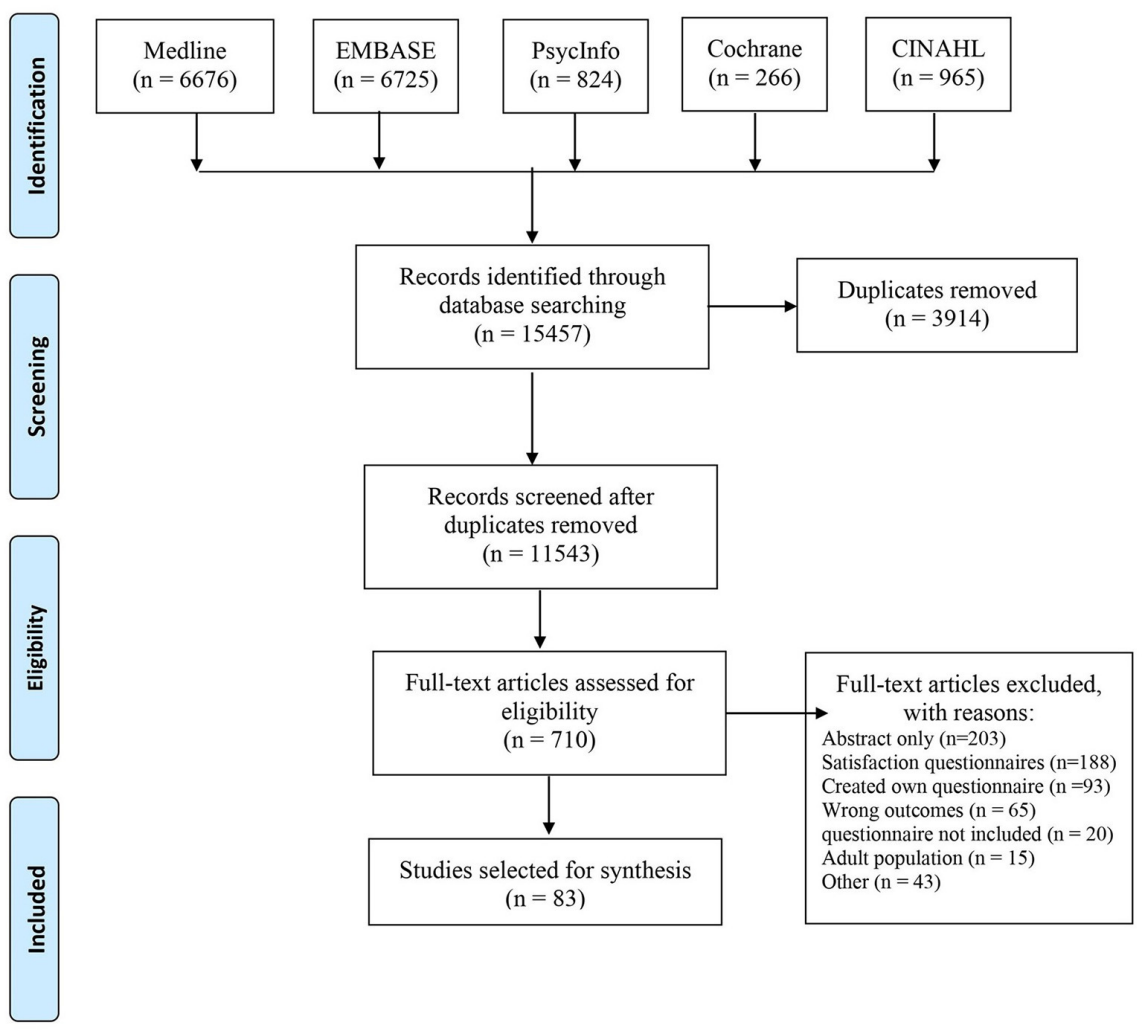
Preferred Reporting Items for Systematic Review and Meta-Analysis (PRISMA) flow diagram of identification and selection process of studies.

The PRISMA flow diagram (Figure 1) summarizes the study selection process. The initial search identified 15,457 articles. After removing duplicates, the title and abstracts of 11,543 articles were screened. Of these, 710 were eligible for full-text review. In total, 83 articles met the inclusion criteria and were included in the analyses. These studies reported on the use of 39 different PREMs in pediatric healthcare settings (Table 1). The gray literature retrieved 10 additional PREMs that are used in clinical practice. Since many PREMs are usually copy-righted by the developers, so we did not contact authors or developer of the surveys for more information.

**Table 1:** Summary of the pediatric patient-reported experience measures.

**No**.	**PREM name**	**Year and country of origin**	**Type (generic/ disease specific)**	**Patient or proxy completion**	**Number of items**	**Number of domains**	**Domain descriptors**
1	Child HCAHPS	2015, United States (10)	Generic	Proxy	62	5	Communication with parent, Communication with child, Attention to safety and comfort, Hospital environment, Global ratings.
2	Children and Young People's Inpatient and Day Case Survey 2014	2014, United Kingdom (11)	Generic	Both	74	10	Presence of pain, Pain relief, Overall experience, Involvement in decisions, Communication on arrival, Communication about care and treatment, Communication before operation/procedure, Communication after operation/procedure, Discharge communication, Advice on post-discharge care.
3	Children's Revised Humane Care Scale (CRHCS)	2019, Finland (12)	Generic	Both	41	6	Professional practice, Information and participation in own care, Cognition of physical needs, Human resources, Pain and apprehension management, Interdisciplinary collaboration.
4	Clinician and Group Consumer Assessment of Healthcare Providers and Systems (CG-CAHPS)	2007, United States (13)	Generic	Proxy	33	N/A	N/A
5	Consultation and Relational Empathy measure (CARE)	2004, United Kingdom (Scotland) (14)	Generic	Both	10	4	Not specified.
6	Consumer Assessment of Health Plan Study (CAHPS) 2.0 Child Core Questionnaire	2014, United States (15)	Generic	Proxy	7	5	Courtesy and respect of office staff, Helpfulness of office staff, Providers' communication skills with parents, Respect shown to parents by providers, Providers' communication skills with children.
7	Consumer Assessment of Health Plans Survey (CAHPS)	2002, United States (16)	Generic	Proxy	18	9	Getting care quickly, Doctor's communication; Health plan customer service, Getting prescription medicines, Getting specialized services, Family centred care-shared decision making, Family centred care-getting needed information, Family centred care-personal doctor.
8	Consumer Assessment of the Healthcare Providers and Systems (CAHPS)	2012, United States (17)	Generic	Proxy	29	9	Care from nurses, Care from doctors, The hospital environment, Experiences in this hospital, Post-discharge overall rating, Understanding care post-discharge, About patient.
9	Disease-specific patient satisfaction questionnaire	2014, Germany (18)	Disease specific (IBS)	Patient	32	N/A	N/A
10	Epilepsy 12	2002, United Kingdom (19)	Disease specific (epilepsy)	Both	18	N/A	N/A
11	EMPATHIC-30	2011, Netherlands (20)	Generic (ICU)	Proxy	30	5	Information, Care and treatment, Organization and coordination of care, Parents and family engagement, Team care (pediatrician and other clinicians involved in the care of the children), Overall score.
12	Evaluation of the Quality of Diabetes Care' (PEQ-D)	2002, Netherlands (21)	Disease specific (diabetes)	Patient	14	N/A	N/A
13	Experience of Service Questionnaire (ESQ)	2002, United Kingdom (22)	Disease specific (mental health)	Both	12	N/A	N/A
14	Family-Provider Relationships Instrument-NICU (FAMPRO-NICU)	2001, United States (23)	Generic (NICU)	Proxy	65	3	Belief-desire, Feelings, Intentions.
15	FCCS (Family Centered Care Survey)	2006, Canada (24)	Generic	Proxy?	20	N/A	N/A
16	GYV (Give Youth a Voice)	2008, Canada (25)	Generic	Patient	56	4	Supportive and respectful relationships, Information sharing and communication, Support of independence, Teen centered services. Note: adapted from MPOC.
17	Inpatient Survey (IS)	2013, United Kingdom (26)	Generic	Patient	86	N/A	N/A
18	McLean Hospital's Perception of Care survey	2002, United States (27)	Generic (inpatient psychiatric care)	Both	20	4	Interpersonal aspects of care, Continuity/coordination of care, Communication/information received from treatment providers, Global evaluation of care.
19	MPOC-20, MPOC-32, MPOC-56	1996, Canada (28)	Generic	Proxy	20, 32, 56	5	Enabling and partnership, providing general information, Providing specific information about the child, Coordinated and comprehensive care, Respectful and supportive care.
20	Mind the Gap	2007, United Kingdom (29)	Generic	Both	22	3	The environment, Care processes, Healthcare provider characteristics.
21	Neonatal Instrument of Parent Satisfaction (NIPS)	1996, Canada (30)	Generic (NICU)	Proxy	27	N/A	N/A
22	NRC Health Patient Survey	2020, United States (31)	Generic	Proxy	20	N/A	N/A
23	P-MISS (Medical Interview Satisfaction Scale)	1986, United States (32)	Generic	Proxy	23	3	Parent communication and child communication, Parent communication and adherence intent, Distress relief and adherence
24	Parent's Perceptions of Primary Care (P3C)	2001, United States (33)	Generic	Proxy	23	6	Continuity of care, Accessibility of care, Contextual knowledge of physicians, Communication skills of physicians, Comprehensiveness of care, Coordination of care.
25	Pediatric Family Satisfaction Questionnaire (PFSQ)	2002, United States (34)	Generic	Proxy	35	3	Hospital services and accommodation, Nursing care, Medical care
26	Pediatric Family Satisfaction-ICU (pFS-ICU)	2001, United States (35)	Generic (ICU)	Proxy	24	5	How did we treat your family member (the patient), Symptom management: how well the ICU staff assessed and treated your child's symptoms, How did we treat you? Information needs, Process of making decisions.
27	PedsQL - Healthcare Satisfaction Generic Module	2005, United States (36)	Generic	Both	26	6	Information, Family inclusion, Communication, Technical skills, Emotional needs, Overall satisfaction.
28	Picker Inpatient Survey	1990's, United States (37)	Generic	Proxy	35	7	Partnership, Overall care, Physical comfort, Information to parents, Confidence and trust, Continuity and transition, Coordination of care.
29	Press Ganey Inpatient Pediatric Survey	1998, United States (38)	Generic	Proxy	38	8	Admission, Nursing care, Tests and treatments; Family and visitors, Child's physician, Discharge, Personal issues, Overall assessment.
30	Press Ganey Medical Practice Survey	United States (39)	Generic	Proxy	29	6	Access to care, Visit processes, Nursing, Care provider, Personal issues, Overall assessment.
31	Press Ganey Patient Satisfaction Survey	United States (40)	Generic	Patient	Not provided	4	Inpatient overall, ED overall, Inpatient speed of admission, ED wait times to treatment
32	Press Ganey Physician Specialties Survey	United States (41)	Generic	Proxy	39	N/A	N/A
33	Press Ganey Satisfaction Survey (unique to each study)	United States (42)	Generic	Proxy	Varies	Varies	Varies
34	Swedish Pyramid Questionnaire (Quality of Patient Care Questionnaire - Parents Version)/ Swedish parent satisfaction questionnaire	2001, Sweden (43)	Generic	Proxy	63	8	Information on illness, Information on routines, Accessibility, Medical treatment, Care processes, Staff attitudes, Parent participation, Staff work environment.
35	The Picker Institute's Neonatal Intensive Care Unit Family Satisfaction survey	2014, United States (44)	Generic (NICU)	Proxy	80	8	Information and education to parents, Environment and visitation policies, Family and infant support by the NICU, Confidence and trust in the NICU, Continuity and transition, Family participation in care, Overall impressions, Coordination of care.
36	The Children's Hospital Boston Inpatient Experience Survey	2013, United States (45)	Generic	Proxy	62	8	Care from nurses, Care from doctors, Doctors/nurses/parents working together, Hospital experiences (procedures, pain management, comfort), Hospital environment, Child's medication, Arrival at and discharge from the hospital, Overall ratings.
37	The national cancer patient experience survey	2010–2014, United Kingdom (46)	Disease Specific (cancer)	Patient	79 (varied each year)	N/A	N/A
38	The patient-reported experience measure (PREM) for children in urgent and emergency care.	2012, United Kingdom (47)	Generic (emergency care)	Both	29	N/A	N/A
39	Young Patient Survey	2004, United Kingdom (48)	Generic	Both	89	9	Respect for patient preferences, Coordination of care, Information and education, Physical comfort, Emotional support, Involvement of family and friends, Continuity and transition, Overall quality of care, Confidentiality and privacy.

### Characteristics of Included Studies

**Figure 2:**
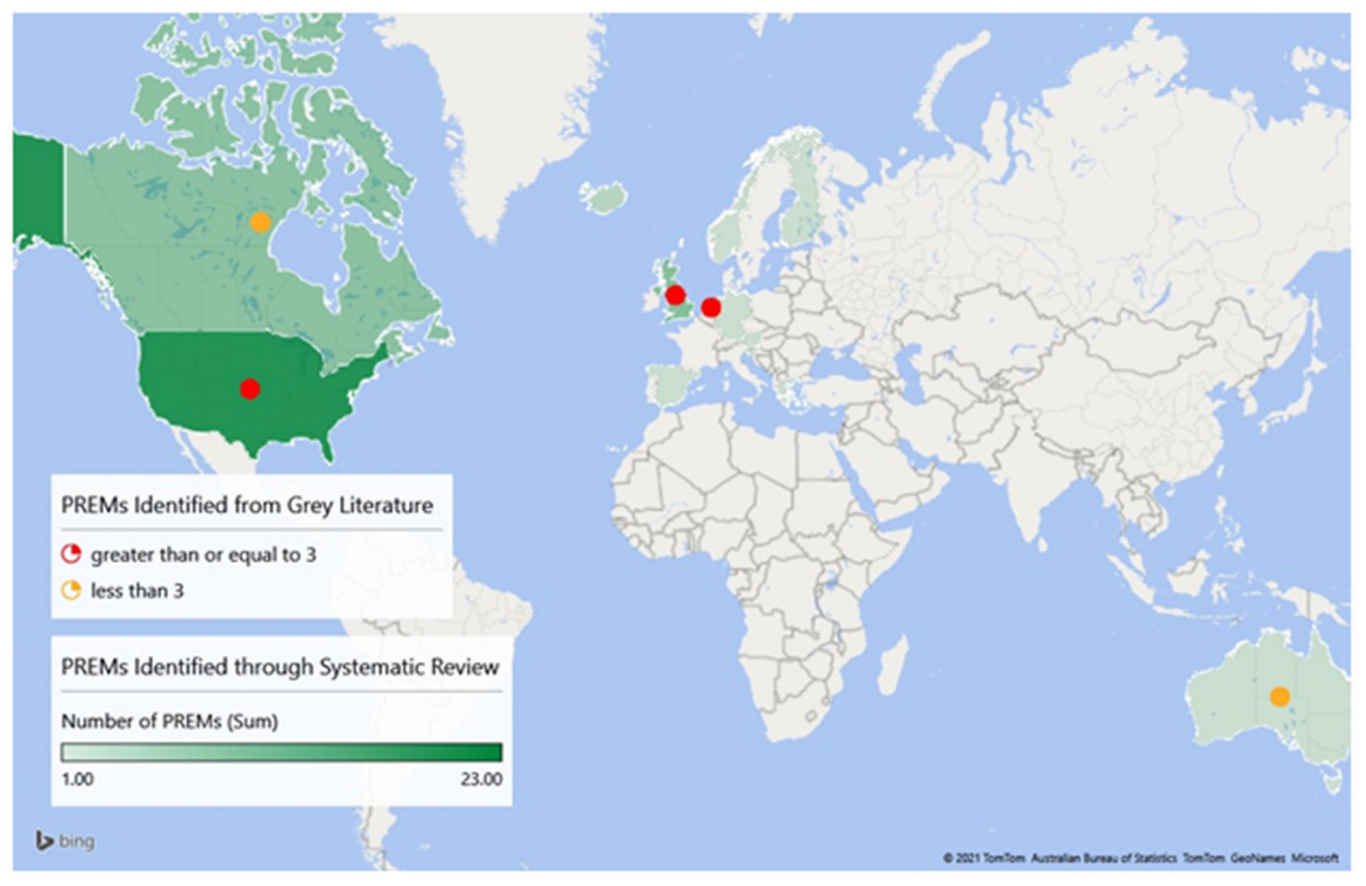
Geographic locations of pediatric PREMs identified through systematic review and gray literature.

The included studies were conducted in 14 countries, including 48 studies in the United States of America, 25 studies in European countries (Austria, Finland, Germany, Greece, Iceland, the Netherlands, Norway, Slovenia, Spain, and the United Kingdom), 8 studies in Canada, and 1 study each in Australia and Singapore. Figure 2 provides an overview of the number of different PREMs that are used per country. Regarding study design, 41 of the included studies used a cross-sectional study design. The remaining study designs include 13 cohort studies, 6 mixed-methods, 6 observational, 3 quasi-experimental, 2 randomized-control trials, 2 quality improvement studies, 2 secondary data analyses, 2 retrospective data studies, and 1 of each of the following study designs: program evaluation, descriptive, longitudinal, case study, and pilot/feasibility studies.

The duration of studies ranged from 2 weeks to 5 years and study populations ranged from 0 to 25 years. Disease of interest varied across studies, although most studies addressed a general disease group. Disease-specific populations included epilepsy, diabetes, asthma, neurological conditions, and juvenile arthritis, among others. The identified studies also presented a diverse range of health care settings, including emergency rooms, NICU and PICUs, inpatient wards, and outpatient ambulatory clinic, among others Overall, paper-pencil was the most common mode of administering PREMs (60.2%), followed by electronic (26.5%), telephone (12.0%), and/or *via* interview (1.2%) modes. The PREMs were completed by proxy only in 60 studies (71.4%), by patient and/or the proxy in 14 studies (16.7%), by only the patient in 7 studies (8.3%), while 3 studies did not specify (3.6%) (one study used two different PREMs that had different methods of completion) (49).

Among the studies, the most commonly used PREMs were the various Press Ganey surveys (50) (*n* = 18), the Measure of Processes of Care (MPOC) (28) (*n* = 15), Child HCAHPS (10) (*n* = 10) and HCAHPS surveys (51) (*n* = 5). Among all the included studies, there was a high amount of variation in the purpose of using PREMs. PREMs were most commonly used to evaluate factors that affected overall patient experience and to assess the experiences after an intervention, most often an improvement in quality of care. A complete overview of the characteristics of the included studies can be found in the Supplement 1.

### PREMs

This extensive review identified 39 previously validated pediatric PREMs (Table 1). These PREMs were developed in 7 countries. The greatest number of PREMs were developed in the United States of America (*N* = 21), followed by the United Kingdom (*N* = 9), Canada (*N* = 4), the Netherlands (*N* = 2), Finland (*N* = 1), Germany (*N* = 1), and Sweden (*N* = 1). Most of the included PREMs were generic (*N* = 34), aiming to measure general experiences of healthcare regardless of the disease or care sector. The included disease specific PREMs (*N* = 5) have been developed to capture the experience of healthcare from patients with inflammatory bowel disease, epilepsy, diabetes, cancer, and mental health conditions.

Of the identified PREMs, 23 were designed to be completed by proxy (59.0%), 10 PREMs (25.6%) could be completed by either or both of the pediatric patient and their caregiver(s), and 6 PREMs (13.4%) had been explicitly developed for completion by the pediatric patients themselves. The PREMs designed for completion by the pediatric patients were Disease-Specific Patient Satisfaction Questionnaire (18), Evaluation of the Quality of Diabetes Care (21), the Give Youth a Voice (25), the Inpatient Survey (26), the Press Ganey Patient Satisfaction Survey (40), and the National Cancer Patient Experience Survey (46).

Among the studies reporting questionnaire characteristics, the number of items ranged from 7 to 89, and the number of domains ranged from 3 to 10. The number of items was not provided for 5 PREMs, and the number of domains was not provided for 12 PREMs. Domains related to communication were most common, such as “communication with parent,” “communication with child,” “communication about care and treatment,” and “provider's communication skills.” Other examples of reported domains include “information,” “respect,” “coordination of care,” “patient and family engagement,” “respectful and supportive care,” and “overall experiences.”

### Quality Assessment

The quality assessment of the included studies is presented in the Supplement 1. The quality of all studies was rated as either fair (40%) or good (60%). Overall, the risk of bias of the included studies was moderate.

### Gray Literature

Ten additional PREMs were identified through the gray literature search (Supplement 2). These PREMs were used to evaluate the experiences of pediatric patients and/or caregivers with daily clinical healthcare in the United States of America, the United Kingdom, the Netherlands, Canada, and Australia. The majority of hospitals in these countries asked patients to share their experiences with the use of PREMs. The gray literature search showed that a variation of PREMs, often self-developed, were used in the hospitals. Some hospitals administered PREMs to all their patients/caregivers, though most hospitals randomly invited recently discharged patients/caregivers to complete PREMs. The modes of administering PREMs identified through gray literature were similar to the ones identified through scientific databases, as listed previously.

## Discussion

In this review, we document the geographic distribution of pediatric PREMs used and quantify the different PREMs administered in clinical care. PREMs are often falsely synonymized with patient reported outcome measures (PROMs) and satisfaction questionnaires, but these three types of questionnaires have distinct purposes and target different elements of patient care. In contrast to PROMs, which assess the patient's health status and measures quality of life, PREMs focus on care processes and their perceived impact on overall patient experience (52). While dissimilar in outlook, PREMs and PROMs are often used in tandem to gather information related to the patient's care experience and outcome contentment. The terms patient satisfaction and patient experience despite being often used interchangeably, are different. Patient experience assesses whether something that should happen in a healthcare setting (such as clear communication with a provider) actually happened or how often it happened. On the other hand, Satisfaction is about whether a patient's expectations about a health encounter were met (52–54). PREMs also differ from patient satisfaction surveys, which relate to patient expectations and often involve a degree of subjectivity that is not seen in PREMs (52, 55–57).

The results of our review demonstrate an international uptake of pediatric PREMs in clinical care, totaling 49 different PREMs, 39 from peer-reviewed articles and 10 from gray literature that were used in 14 developed countries spanning four continents. While administered in 14 different countries, the development of these PREMs only occurred in seven, with the greatest heterogeneity in both pediatric PREM development and implementation occurring in the United States (21), followed by the United Kingdom (9). While primarily utilized for quality improvement purposes, various research groups implemented pediatric PREMs to gauge how the responses varied between patient populations or between the patients and their family caregivers.

Measuring patient and family experience has a critical role in informing PFCC. Previous studies have explored the development and psychometric evaluation of PREMs, assessed their validity and reliability, and compared different PREM instruments for their respective utilization (58–60). Studies have also noted differences between proxy ratings, usually coming from a family caregiver, and the ratings of a patient themselves, where the patient tends to provide lower rating regarding their own experiences of care (49, 61). Additionally, there exists a paucity of information regarding the use of pediatric PREMs, and their type (i.e., generic, disease-specific, health-setting-specific), as well as their purpose and impact on quality of care in clinical practice. These findings can be used to inform PFCC initiatives at a system-level, helping to achieve the Triple Aim and supporting the learning health system paradigm (62, 63).

Additionally, research has acknowledged the correlation between PREM-implementation, the establishment of the PFCC, and the promotion of quality improvement initiatives (64). While this information is accepted in the context of adult PREMs, much less research exists regarding the implementation and assessment of pediatric PREMs (65). This study will inform future work in the area of PREM implementation in pediatric care.

The identified PREMs feature important domains addressing PFCC concepts such as shared-decision making and respecting patient values. A main gap identified in our review suggests that the use of disease-specific PREMs warrants more attention, with only five of the validated PREMs being disease-specific. Even among studies conducted in disease-specific settings, generic PREMs were more often chosen over an appropriate disease-specific tool. This may be related to the versatility and applicability of generic PREMs in more healthcare settings compared to disease-specific PREMs. However, disease-specific PREMs issues more specific to the corresponding disease. For example, MPOC (28) is a validated PREM commonly used for children with variety of neurodevelopmental disabilities or maxillofacial disorders. MPOC assesses family caregiver's perception of the care that their children receive at rehabilitation treatment centers, and thus can provide a better contextual understanding of patient experience specifically related to those clinical conditions. Therefore, future research examining why disease-specific PREM use and development is lacking should be explored. Additionally, while all included studies discussed the utility of using these pediatric PREMs, few examined the practicality of implementing them (66–68). Future research examining the capacity of hospitals and physicians to incorporate these measures into clinical care is needed to pragmatically assess the likelihood of pediatric PREM administration.

A significant strength of this systematic review is the inclusion of gray literature. As this review aimed to explore the range of pediatric PREMs currently in use, gray literature sources provided an exploration of real-world PREM implementation in pediatric healthcare settings around the world. We also incorporated the perspectives of international researchers with expertise in the topics of PREMs and PROMs. This bolstered the knowledge and experience of the research team and allowed for the inclusion of different perspectives on PREM implementation from different countries.

Despite being successful in identifying the number of pediatric PREMs currently in use, this review was not without limitations. Regarding gray literature, the information about the PREMs and their implementation were often not explicitly described on hospital websites, meaning we could only provide a global description of these PREMs. PREMs created in or translated to different languages or cultural contexts may have not been available in a language that the reviewers could understand, and therefore those studies were excluded. Furthermore, the inclusion criteria of “high-income countries” potentially limited the scope of this study by geographically restricting the results. Lastly, because of the interchangeable use of the terms “experience” and “satisfaction,” it is possible that due to the phrasing of study surveys, some PREMs were inadvertently excluded. However, the likelihood of this occurring was minimized due to the continual implementation of dual reviewers and the inclusion of the terms “satisfaction” and “satisfaction survey” in our initial search strategy.

The objective of this systematic review was to identify pediatric PREMs and their use in care settings. Although there are tools like the COSMIN Checklist to critically appraise the validity and reliability of PROs (PROMs and PREMs), there are no such standard tools to evaluate the strengths and weaknesses of PREMs. Moreover, evaluating these measures for their strengths and weaknesses would be subjective and context specific. Therefore, this systematic review did not evaluate the strengths and weaknesses of the PREMs, but further studies focused on assessing the strengths and weaknesses of individual PREMs may be warranted in the future.

## Conclusion

This systematic review details the international use of pediatric PREMs in different pediatric clinical settings and provides an overview of the current validated pediatric PREMs available for use. The findings of this review can guide health administrators and researchers to use appropriate PREMs to implement PFCC in pediatric settings. In most of the studies included in this review, the usefulness of pediatric PREMs was highlighted. However, future additional research into the views of implementing PREMs held by clinical practitioners and patients and their families is warranted to best gauge the practicality of widespread pediatric PREM implementation.

## Data Availability Statement

The original contributions presented in the study are included in the article/Supplementary Material, further inquiries can be directed to the corresponding author/s.

## Author Contributions

MS, LH, SB, SA, EG, LT, and HO contributed to the overall rationale and design of the review. ND provided expert input on developing search strategy. SB, MZ, SR, SA, and LT assessed studies for eligibility against inclusion and exclusion criteria including gray literature and assessed risk of bias and also extracted the data and synthesized results. SB, MZ, SR, and SA led the drafting of the manuscript. MS, LH, SB, SA, EG, LT, HO, MZ, and SR contributed to the subsequent drafts of the manuscript. All authors contributed to the article and approved the submitted version.

## Funding

SB received financial support in the form of graduate studentship from Alberta Children's Hospital Research Institute. MZ received financial support in the form of summer research studentship from Alberta Innovates.

## Conflict of Interest

EG was employed by Evidera. The remaining authors declare that the research was conducted in the absence of any commercial or financial relationships that could be construed as a potential conflict of interest.

## Publisher's Note

All claims expressed in this article are solely those of the authors and do not necessarily represent those of their affiliated organizations, or those of the publisher, the editors and the reviewers. Any product that may be evaluated in this article, or claim that may be made by its manufacturer, is not guaranteed or endorsed by the publisher.

## Abbreviations

Child HCAHPS: The Child Hospital Consumer Assessment of Healthcare Providers and Systems
IHI: Institute for Healthcare Improvement
MeSH: Medical Subject Headings
MPOC: Measure of Process of Care
PFCC: Patient and Family-Centered Care
PREM: Patient-Reported Experience Measure
PRISMA: Preferred Reporting Items for Systematic Review and Meta-Analysis
PROM: Patient-Reported Outcome Measure.
