# Supplementary material for: Use of Patient-Reported Experience Measures in Pediatric Care: A Systematic Review
Source: Front Pediatr. 2021 Dec 20;9:753536. doi: 10.3389/fped.2021.753536 (PMC8721567; doi:10.3389/fped.2021.753536)
Supplement: Supplementary file 1 [file Table_1.DOCX]

**Supplement 1:** Overview of the characteristics of included studies

| **No.** | **Author(s), year, country of study** | **Study objective** | **Study design** | **Study duration** | **Disease group** | **Study setting** | **Patients’ age group (yrs)** | **Number of participants completing PREMs** | **PREM used in the study** | **Person completing the survey** | **Administration method** | **Quality score** |
| --- | --- | --- | --- | --- | --- | --- | --- | --- | --- | --- | --- | --- |
| **1** | Krugman, Scott D; Suggs, Adrienne; Photowala, Hasnain Y; Beck, Adam, 2007, USA^1^ | To measure the effects of combining an emergency department and pediatric inpatient unit. | Interventional study | 18 months | General | Emergency department and inpatient care units | NR | NR | Press Ganey patient satisfaction survey | Patient | NR | Fair |
| **2** | Matziou, Vasiliki; Boutopoulou, Barbara; Chrysostomou, Anthi; Vlachioti, Efrosini; Mantziou, Theodora; Petsios, Konstantinos, 2011, Greece[^2^](https://www.zotero.org/google-docs/?jCvIjV) | To record parental satisfaction with care during hospitalization and assess its determinants. | Descriptive, non-experimental | 4 months | General | Surgical and inpatient care units | < 14 | 206 | Swedish Pyramid Questionnaire (Quality of Patient Care Questionnaire - Parents Version) | Proxy | In-person interview | Good |
| **3** | Williams, Fiona; McCafferty, Aileen; Dunkley, Colin; Kirkpatrick, Martin, 2018, UK[^3^](https://www.zotero.org/google-docs/?G6GV7R) | To survey patients experiences with epilepsy in the UK. | Cross- sectional | 12 months | Epilepsy | Audit units | NR (many were 5-15) | 2335 | "Epilepsy 12", patient satisfaction experience questionnaire | Either the patient and proxy | NR | Fair |
| **4** | Voos, Kristin C; Ross, Gail; Ward, Mary J; Yohay, Anne-Lise; Osorio, Snezana Nena; Perlman, Jeffrey M, 2011, USA[^4^](https://www.zotero.org/google-docs/?7Z9xmH) | To evaluate the satisfaction of care providers and parents following practice changes. | Quasi- experimental | 10 months | General | NICU | Neonates | 28 | Neonatal Instrument of Parent Satisfaction (NIPS[26]) | Proxy | Paper and electronic | Good |
| **5** | Nichol J.R.; Fu R.; French K.; Momberger J.; Handel D.A., 2016, USA[^5^](https://www.zotero.org/google-docs/?xg5zPh) | To determine the key variables found in 3 separate PREMs. | Retrospective cohort | 3 years 6 months | General | Emergency department | <20 | 810 | Press Ganey patient satisfaction surveys | Either the patient or proxy | Paper and electronic | Fair |
| **6** | Sigurdardottir, Anna Olafia; Garwick, Ann W.; Svavarsdottir, Erla Kolbrun, 2017, Iceland [^6^](https://www.zotero.org/google-docs/?jC709Y) | To assess parental predictors of healthcare satisfaction throughout an Icelandic hospital. | Cross- sectional | 2 years | General | Children’s hospital (neonatal, emergency, day, and outpatient units) | ≤18 | 219 | PedsQL - Healthcare Satisfaction Generic Module | Proxy | Paper and electronic | Good |
| **7** | Timmer, Antje; Peplies, Jenny; Westphal, Max; Kaltz, Birgit; Ballauff, Antje; Clasen, Martin; Laass, Martin W; Koletzko, Sibylle, 2017, Germany and Austria[^7^](https://www.zotero.org/google-docs/?dskzmw) | To assess patients’ experiences with transitioning between pediatric and adult care settings. | Cross- sectional | NR | Irritable Bowel Diseases | Varied | 15-25 | 583 | "Disease- specific patient satisfaction questionnaire" | Patient | Paper | Good |
| **8** | Sleath, Betsy; Ayala, Guadalupe X; Washington, Deidre; Davis, Stephanie; Williams, Dennis; Tudor, Gail; Yeatts, Karin; Gillette, Chris, 2010, USA[^8^](https://www.zotero.org/google-docs/?XJM8oL) | To examine the correlation between parental ratings of participatory decision making and satisfaction with their pediatric asthma visits. | Cross- sectional | NR | Asthma | Pediatric Practices | 8-16 | 320 | P-MISS (Medical Interview Satisfaction Scale) | Patient | Paper | Good |
| **9** | De Wit M.; Delemarre-van De Waal H.A.; Bokma J.A.; Haasnoot K.; Houdijk M.C.; Gemke R.J.; Snoek F.J., 2008, Netherlands [^9^](https://www.zotero.org/google-docs/?TOzE88) | To analyze the effects of discussing health-related quality of life (HRQoL) in pediatric patients with type 1 diabetes. | Randomized control trial | 1 year | Type 1 Diabetes | Outpatient clinics | 13-17 | 81 | Patients' Evaluation of the Quality of Diabetes Care (PEQ-D) | Patient | NR | Good |
| **10** | Cohen, Eyal; Austin, Janice; Weinstein, Michael; Matlow, Anne; Redelmeier, Donald A, 2008, Canada[^10^](https://www.zotero.org/google-docs/?F3jzQx) | To assess quantitatively and qualitatively the care provided to isolated versus non-isolated patients. | Prospective observational | 3 months | General | Inpatient unit | NR | 65 | Pediatric Family Satisfaction Questionnaire (PFSQ) | Proxy | Paper | Good |
| **11** | Galbraith, Alison A; Semura, Jeanne; McAninch-Dake, Becky; Anderson, Nancy; Christakis, Dimitri A, 2004, USA[^11^](https://www.zotero.org/google-docs/?WHZjzH) | To determine whether a correlation exists between emergency department use and perceived lags in accessing acute care. | Cross- sectional | 5 months | General | Emergency department inpatient unit | 10-14 | 5142 | CAHPS | Proxy | Paper and telephone | Good |
| **12** | Adams, E Kathleen; Ketsche, Patricia; Zhou, Mei; Minyard, Karen, 2008, USA[^12^](https://www.zotero.org/google-docs/?bbNQNq) | To track patient experience and satisfaction with a new insurance program (PeachCare). | Cross- sectional | 3 years | General | Varied | ≤18 | 2089 | CAHPS | Proxy | Paper and telephone | Good |
| **13** | Agra Tuñas, Maria del Carmen; Pérez Várela, Fátima; Bello Rama, Eva; Mato Guerra, Paula; Calviño Vieito, Nuria; Garrudo Díaz, Rubén; Pérez Rivas, Manuela; Rodríguez Núñez, Antonio, 2018, Spain[^13^](https://www.zotero.org/google-docs/?euhYeQ) | To evaluate perceived quality of care in a PICU from the viewpoint of a patient's relative. | Cross- sectional | 1 year 1 month | General | Intensive care unit | 1month -16 | 181/308 | EMPATHIC- 30 questionnaire | Proxy | Paper and electronic | Fair |
| **14** | Mah, Jean K; Tough, Suzanne; Fung, Thomas; Douglas-England, Kathleen; Verhoef, Marja, 2006, Canada[^14^](https://www.zotero.org/google-docs/?MHOaWQ) | To assess patient responsiveness to PREMs, the association between patient care experience and HRQoL, and whether patient and parent responses differ. | Cross-  sectional | 4 months | Neurologic diseases | Outpatient care | 12-18 | 104 | Family Centered Care Survey (FCCS), MPOC-20, Give Youth a Voice (GYV) | Both the patient and participant | NR | Good |
| **15** | Mason, Sheila L; Chike-Harris, Katherine E; Gyr, Bettina M; Johnson, Emily, 2019, USA[^15^](https://www.zotero.org/google-docs/?mM25ee) | To evaluate whether implementing a discharge facilitator will improve the discharge process. | Retrospective cohort | Pre-intervention: 3 months, Post-intervention: 3 months | Orthopedic diseases | Orthopedic surgery ambulatory clinic (inpatient and outpatient) | 8months -18 | 219 | Press Ganey Satisfaction Survey | Proxy | Electronic | Fair |
| **16** | Stefansdottir, Sara; Thora Egilson, Snaefridur, 2016, Iceland[^16^](https://www.zotero.org/google-docs/?EavZEF) | To assess parental and therapist perceptions of FCS, the factors that influence parental satisfaction with care, and how parents understood the service. | Mixed methods | 1 year | Physical rehabilitation | Outpatient care | 0-18 | 236 | MPOC-32 | Proxy | Electronic | Fair |
| **17** | Siebes, R C; Wijnroks, L; Ketelaar, M; van Schie, P E M; Gorter, J W; Vermeer, A, 2007, Netherlands[^17^](https://www.zotero.org/google-docs/?Oznrmh) | To describe parental involvement in their child’s rehabilitation, their satisfaction with treatment quality, and ideas that they have pertaining to increasing parental involvement in rehabilitation. | Longitudinal mixed methods | Phase 1:1 year 7 months, Phase 3: 6 months | Physical rehabilitation | Outpatient care | 1-20 | 679 | MPOC-56 | Proxy | NR | Fair |
| **18** | Van Riper M., 2001, USA[^18^](https://www.zotero.org/google-docs/?TtuXh6) | To assess maternal perceptions of their patient-provider relationship and how this relates to patient well being. | Cross- sectional | NR | Prematurity | NICU | NR | 55 | Family-Provider Relationships Instrument- NICU (FAMPRO- NICU) | Proxy | Paper | Fair |
| **19** | Nieman, Carrie L; Benke, James R; Ishman, Stacey L; Smith, David F; Boss, Emily F, 2014, USA[^19^](https://www.zotero.org/google-docs/?A7khBU) | To evaluate race and insurance-based disparities in PREM participation. | Cross- sectional | 7 months | Otolaryngologic diseases | Tertiary otolaryngology clinic | 0-17 | 130 | Press Ganey Medical Practice Survey | Proxy | Paper and electronic | Good |
| **20** | Wells, R D; Dahl, B; Wilson, S D, 2001, USA[^20^](https://www.zotero.org/google-docs/?gAi6dC) | To assess differences in care satisfaction between patients and their primary care provider. | Prospective cohort | 9 months | Asthma, bronchitis, gastroenteritis, pneumonia | Inpatient care unit | 1month -18 | 181 | Press-Ganey Physician Satisfaction Scale | Proxy | Paper and telephone | Good |
| **21** | Toomey, Sara L; Elliott, Marc N; Zaslavsky, Alan M; Klein, David J; Ndon, Sifon; Hardy, Shannon; Wu, Melody; Schuster, Mark A, 2017, USA[^21^](https://www.zotero.org/google-docs/?opfkgc) | To measure the performance of different hospitals in a national field test. | Observational study | 15 months | General | Inpatient care units | 0-17 | 17727 | Child HCAHPS | Proxy | Paper and telephone | Fair |
| **22** | Terwiel, M; Alsem, M W; Siebes, R C; Bieleman, K; Verhoef, M; Ketelaar, M, 2017, Netherlands [^22^](https://www.zotero.org/google-docs/?vzV6jQ) | To assess the importance of each PREM item related to FCS. | Observational study | NR | Cerebral palsy | Rehabilitation centers (outpatient care) | 3-9 | 175 | MPOC-56 | Proxy | NR | Fair |
| **23** | Williams, Geraint; Pattison, Giles; Mariathas, Chrishan; Lazar, Joanna; Rashied, Muhammad, 2011, UK[^23^](https://www.zotero.org/google-docs/?orIEBN) | To pinpoint aspects of patient care that could be improved to increase patient satisfaction. | Prospective observational | 10 months | Orthopedic diseases | Inpatient care units | 2-6 | 104 | Swedish parent satisfaction questionnaire | Proxy | NR | Good |
| **24** | Nieman, Carrie L; Benke, James R; Boss, Emily F, 2015, USA[^24^](https://www.zotero.org/google-docs/?9FykZ1) | To record patient satisfaction with clinical care and stratify results according to patient race, ethnicity, or SES. | Cross- sectional | 1 year | General | Outpatient surgical care | 0-17 | 527 | Press Ganey Medical Practice Survey | Proxy | Paper and electronic | Fair |
| **25** | Tothy, Alison S; Limper, Heather M; Driscoll, James; Bittick, Nicholas; Howell, Michael D, 2016, USA[^25^](https://www.zotero.org/google-docs/?QoOVZb) | To assess the effects of implementing a package of improvement activities on patient care satisfaction. | Cross- sectional | 11 months | General | Emergency department inpatient care | NR | 352 | Press Ganey Satisfaction Survey | Both the patient and proxy | Paper | Fair |
| **26** | Tilly-Gratton A.; Nadon M.A.; Houle A.; Pelaez S.; Ducharme F.M., 2018, Canada[^26^](https://www.zotero.org/google-docs/?mg7pUt) | To investigate patient satisfaction with clinical care and identify modifiable factors that may increase treatment adherence. | Mixed methods | NR | Asthma | Outpatient clinic | 1-17 | 24 | P-MISS (Medical Interview Satisfaction Scale) | Proxy | NR | Good |
| **27** | Sonneveld, H. M.; Strating, M. M. H.; van Staa, A. L.; Nieboer, A. P., 2013, Netherlands[^27^](https://www.zotero.org/google-docs/?YR1j49) | To examine the perceptions of parents, providers, and patients on transitional care experiences and to determine the extent to which perspective differences were disease specific. | Cross- sectional | NR | Type 1 diabetes, juvenile rheumatoid arthritis, neuromuscular disorder | Outpatient clinic | 12-25 | 319 | Mind the Gap | Both the patient and proxy | Paper and electronic | Fair |
| **28** | Siebes, R C; Wijnroks, L; Ketelaar, M; van Schie, P E M; Vermeer, A; Gorter, J W, 2007, Netherlands[^28^](https://www.zotero.org/google-docs/?shm6zO) | To assess the stability of the MPOC survey 1 year post implementation. | Longitudinal survey | 1 year 1 month | Chronic diseases | Rehabilitation centers (outpatient care) | 1-20 | 205 | MPOC-56 | Proxy | Paper | Good |
| **29** | Locke, Robert; Stefano, Mariane; Koster, Alex; Taylor, Beth; Greenspan, Jay, 2011, USA[^29^](https://www.zotero.org/google-docs/?ab6HJP) | To assess parental satisfaction with the care provided in the emergency department. | Retrospective cohort | 6 months | General | Emergency department outpatient care | IQ range 1-10 | 456 | Press Ganey Satisfaction Survey | Proxy | NR | Fair |
| **30** | Camilon, P Ryan; Levi, Jessica R; Carrion, Rose A; Josephson, Gary D, 2019, USA[^30^](https://www.zotero.org/google-docs/?Lu2Xam) | To identify PREM items that are most closely correlated with the “likelihood of recommending practice”. | Retrospective cohort | 1 year | Otolaryngologic diseases | Outpatient care | NR | 3401 | Press Ganey Outpatient Medical Practice Survey | NR | NR | Fair |
| **31** | Shaw, K L; Southwood, T R; McDonagh, J E; British Society of Paediatric and Adolescent Rheumatology, 2007, UK[^31^](https://www.zotero.org/google-docs/?XaIWN0) | To document the expectations and satisfaction of parents and patients with their transitional care experiences prior to and following the implementation of a related programme. | Cohort | NR | Juvenile idiopathic arthritis | Outpatient care | 11-18 | 308 | Mind the Gap Scale | Both the patient and proxy | NR | Good |
| **32** | Shaw, K L; Watanabe, A; Rankin, E; McDonagh, J E, 2014, UK[^32^](https://www.zotero.org/google-docs/?nFBClh) | To evaluate transition care quality through examination of patient adherence to guidelines and whether adherence was associated with improved experience. | Cross- sectional | 2 weeks | Chronic diseases | Outpatient tertiary care | 11-21 | 787 | Mind the Gap Scale | Both the patient and proxy | Paper | Fair |
| **33** | Shevell, Michael; Oskoui, Maryam; Wood, Ellen; Kirton, Adam; Van Rensburg, Esias; Buckley, David; Ng, Pamela; Majnemer, Annette, 2019, Canada[^33^](https://www.zotero.org/google-docs/?PeI3Z4) | To describe characteristics of children with cerebral palsy and factors that impact parent perceptions of FCS. | Cross- sectional | 4 years | Neurodevelopmental disabilities (cerebral palsy) | Rehabilitation centers (outpatient care) | Infants | 312 | MPOC-56 | Proxy | Paper | Good |
| **34** | Singh, Suprit C; Sheth, Raj D; Burrows, James F; Rosen, Paul, 2016, USA[^34^](https://www.zotero.org/google-docs/?tWPAbE) | To identify key factors in facilitating overall satisfaction with ambulatory neurological care. | Cross- sectional | 3 years | Neurologic disease | Ambulatory care units | NR | 2890 | Press Ganey Satisfaction Survey | Proxy | Paper and electronic | Good |
| **35** | Singleton I.M.; Garfinkel R.; Temkit H.; Belthur M.V., 2020, USA[^35^](https://www.zotero.org/google-docs/?EMGmwz) | To determine which factors impact parental satisfaction with their care provider in an outpatient clinic. | Cross- sectional | 1 year 8 months | Orthopedic diseases | Orthopedic outpatient clinic | Mean age of 6 | 122 | Clinician and Group Consumer Assessment of Healthcare Providers and Systems (CG-CAHPS) | Proxy | Paper | Fair |
| **36** | Sng, Qian Wen; Kirk, Angela H P; Buang, Siti Nur Hanim; Lee, Jan Hau, 2017, Singapore[^36^](https://www.zotero.org/google-docs/?LPWhMC) | To assess whether different parental satisfaction scores in a PICU are related to differences in ethnicity and to isolate areas of the PICU that need improvement. | Prospective observational | 1 year | General | PICU | Median age of 3 | 206 | EMPATHIC- 30 | Proxy | Paper | Good |
| **37** | Schreiber, Joseph; Benger, Jennifer; Salls, Joyce; Marchetti, Gregory; Reed, Lindsey, 2011, USA[^37^](https://www.zotero.org/google-docs/?JMM1nP) | To evaluate parental perceptions regarding the amount of FCC exhibited at the rehabilitation facility. | Mixed methods | NR | Neurodevelopmental diseases | Rehabilitation centers (outpatient care) | 1-26 | 246 | MPOC-20 | Proxy | Paper | Fair |
| **38** | Rahi J.S.; Manaras I.; Tuomainen H.; Lewando Hundt G., 2005, UK [^38^](https://www.zotero.org/google-docs/?7tq2tD) | To consolidate information on the needs and experiences of parents whose children have recently been diagnosed with an ophthalmic disorder. | Cross- sectional + qualitative | 2 years | Ophthalmic disorders | Tertiary clinical practice | Mean age of 2 | 147 | MPOC-56 | Proxy | Paper | Good |
| **39** | Rahi, Jugnoo S; Manaras, Irene; Tuomainen, Helena; Hundt, Gillian Lewando, 2004, UK [^39^](https://www.zotero.org/google-docs/?FmRFvr) | To monitor changes in the health service experiences of parents and care providers following the introduction of a key worker service. | Cross- sectional + qualitative | 1 year | Ophthalmic disorders | Tertiary referral center of pediatric ophthalmology | Mean age of 2 | 147 | MPOC-56 | Proxy | Paper | Good |
| **40** | Hargreaves, Dougal S; McDonagh, Janet E; Viner, Russell M, 2013, UK[^40^](https://www.zotero.org/google-docs/?F9PaTn) | To assess the association between the PREMs criteria and reported patient satisfaction in national inpatient surveys. | Cross- sectional | NR | General | Inpatient care unit | 12-17 (YPS); 16-19 (IS) | 7657 (YPS); 988 (IS) | Young Patient Survey (YPS); Inpatient Survey (IS) | Patient | Paper | Fair |
| **41** | Hargreaves, Dougal S; Sizmur, Steve; Pitchforth, Jacqueline; Tallett, Amy; Toomey, Sara L; Hopwood, Bridget; Schuster, Mark A; Viner, Russell M, 2018, UK[^41^](https://www.zotero.org/google-docs/?AJ3V9J) | To quantify the number of patients that completed the CYP PREM portion on their own and how their responses resemble their parents' experience. Secondly, CYP responses were separated and compared according to how they were completed (by the child, parent, or together). | Cross- sectional | 2 months | General | Inpatient care unit | 8-15 | 6204 | Children and Young People’s Inpatient and Day Case Survey 2014 | Both the patient and the proxy | Paper | Good |
| **42** | Myrhaug, Hilde Tinderholt; Jahnsen, Reidun; Ostensjo, Sigrid, 2016, Norway [^42^](https://www.zotero.org/google-docs/?BbcoSj) | To assess the perceptions of parents and patients on the family-centeredness of their care. This was then compared to the extent of parental involvement, child well being, and parental satisfaction. | Cross- sectional | 2 months | Cerebral palsy | Primary clinical practice | <6 | 121 | MPOC-20 | Proxy | Paper | Fair |
| **43** | Nagarajan, Neeraja; Rahman, Sydur; Boss, Emily F, 2017, USA[^43^](https://www.zotero.org/google-docs/?ZG7t2I) | To characterize the connection between patient race and their satisfaction with their care. | Cross- sectional | 1 year | Inpatients | Tertiary inpatient care unit | <18 | 904 | Press Ganey Inpatient Pediatric Survey | Proxy | Paper and electronic | Fair |
| **44** | Peng, Frederick B; Burrows, James F; Shirley, Eric D; Rosen, Paul, 2018, USA[^44^](https://www.zotero.org/google-docs/?f9KbpE) | To identify the primary factors involved in orthopedic patient satisfaction. | Cross- sectional | 3 years | Orthopedic diseases | Orthopedic outpatient care | NR | 6195 | Press Ganey Satisfaction Survey | Proxy | Paper and electronic | Fair |
| **45** | Petitgout, Janine M, 2015, USA[^45^](https://www.zotero.org/google-docs/?vOvJx7) | To monitor changes in patient satisfaction following the implementation of a quality improvement initiative. | Case study | NR | General | Tertiary inpatient care unit | <21 | NR | Press Ganey Satisfaction Survey | Proxy | NR | Fair |
| **46** | Kemp, Kyle A; Ahmed, Sadia; Quan, Hude; Johnson, David; Santana, Maria J,, 2018, Canada[^46^](https://www.zotero.org/google-docs/?W1aQmG) | To illustrate the provincial uptake of the Child HCAHPS survey in Alberta. | Cross- sectional | 1 year 6 months | General | Inpatient care unit | <17 | 3389 | Child HCAHPS | Proxy | Telephone | Good |
| **47** | Kleinsorge, Christy A; Roberts, Michael C; Roy, Kimberlee M; Rapoff, Michael A, 2010, USA[^47^](https://www.zotero.org/google-docs/?V4eYs4) | To determine how well a primary care training facility met primary care standards and how satisfied the patients were with their care. | Program evaluation | NR | General | Primary clinical care | 2-18 | 102 (P3C); 105 (CSQ-8) | Parent’s Perceptions of Primary Care (P3C); CAHPS 2.0 Child Core Questionnaire | Proxy | Paper | Fair |
| **48** | Knapp, Caprice; Madden, Vanessa; Sloyer, Phyllis; Shenkman, Elizabeth, 2012, USA[^48^](https://www.zotero.org/google-docs/?SthC9s) | To examine the effects of introducing an integrated care system on the patients’ perception of care quality and their satisfaction with their child’s treatment. | Quasi- experimental | 2 years | General | Varied | 1-21 | 1727 | Consumer Assessment of Health Plans Survey (CAHPS) | Proxy | Telephone | Good |
| **49** | Knox V.; Menzies S., 2005, UK[^49^](https://www.zotero.org/google-docs/?xZwWTv) | To record the experiences of families receiving therapy, and their perceptions of their quality of care. | Cross- sectional | 1 year | Cerebral palsy | Tertiary clinical care | <18 | 72 | MPOC-56 | Proxy | Paper | Good |
| **50** | Kuo, Dennis Z.; Sisterhen, Laura L.; Sigrest, Ted E.; Biazo, James M.; Aitken, Mary E.; Smith, Christopher E., 2012, USA[^50^](https://www.zotero.org/google-docs/?fQQdaM) | To assess the effects of family centered rounds on improved family experiences and health care utilization. | Prospective cohort | 1 year 3 months | General | Inpatient care unit | <12 | 97 | Consumer Assessment of Healthcare Providers and Systems measures | Proxy | Telephone | Good |
| **51** | Koves I.H.; Boucher A.; Ismail D.; Donath S.; Cameron F.J., 2008, Australia[^51^](https://www.zotero.org/google-docs/?ZFAetp) | To correlate parental or patient satisfaction with better patient outcomes, improved health literacy, and elevated HRQoL. | Cross- sectional | 2 months | Diabetes mellitus | Outpatient care | 5-18 | 217 | Evaluation of the Quality of Diabetes Care’ (PEQD) | Both the patient and the proxy | Paper | Good |
| **52** | Miceli PJ; Clark PA, 2005, USA[^52^](https://www.zotero.org/google-docs/?6memCg) | To document the experiences of hospitalized pediatric patients. | Retrospective database study | 1 year | General | Inpatient care unit | <21 | 50446 | Press Ganey Pediatric Inpatient Survey | Proxy | Paper | Fair |
| **53** | Janhunen, Katja; Kankkunen, Päivi; Kvist, Tarja, 2019, Finland [^53^](https://www.zotero.org/google-docs/?TxofDv) | To compare the experiences and satisfaction of patients and parents in the emergency department, and to identify key factors that predict satisfaction. | Cross- sectional | 6 months | General | Emergency department | 7-16 | 196 | Children’s Revised Humane Care Scale (CRHCS) | Both the patient and the proxy | Paper | Good |
| **54** | Lee, Brian; Hollenbeck-Pringle, Danielle; Goldman, Victoria; Biondi, Eric; Alverson, Brian, 2019, USA[^54^](https://www.zotero.org/google-docs/?1l3TGb) | To assess the accuracy of the child HCAHPS survey in reflecting patient experiences and to determine whether external factors were influencing survey responses. | Retrospective cohort study | 1 year | General | Inpatient care unit | <18 | 363 | Child HCAHPS (C-HCAHPS) | Proxy | Paper | Fair |
| **55** | Hurtubise, Karen; Shanks, Robin; Benard, Laura, 2017, Canada[^55^](https://www.zotero.org/google-docs/?OL8VZD) | To evaluate patient satisfaction with a new orthopedic physiotherapy-led clinic. | Pilot/ feasibility study | 1 year 10 months | Cerebral palsy | Outpatient clinic | NR | 50 | MPOC-20 | Proxy | Paper | Fair |
| **56** | Harder, Valerie S; Krulewitz, Julianne; Jones, Craig; Wasserman, Richard C; Shaw, Judith S, 2016, USA[^56^](https://www.zotero.org/google-docs/?BhBbh3) | To assess whether 2 different aspects of care are associated with improved patient experience, and whether this association changes depending on the type of practice. | Cross- sectional | 2 years | General | Primary clinical care | <18 | 2599 | Consumer Assessment of Health care Providers and Systems (CAHPS). | Proxy | Paper | Fair |
| **57** | Groleger Srsen, Katja; Vidmar, Gaj; Socan, Gregor; Zupan, Anton, 2014, Slovenia [^57^](https://www.zotero.org/google-docs/?W1BW4s) | To evaluate clinical processes of care and identify provider characteristics that are correlated with increased patient satisfaction. | Cross- sectional | NR | Chronic diseases | Inpatient and outpatient care | <18 | 235 | MPOC-20 | Proxy | NR | Good |
| **58** | Hall, Allyson G; Landry, Amy Yarbrough; Lemak, Christy Harris; Boyle, Erin L; Duncan, R Paul, 2014, USA[^58^](https://www.zotero.org/google-docs/?vtTGrx) | To assess how patient and parent satisfaction fluctuates with the child’s health status and their prescribed treatment plan. | Cross- sectional | 3 years | General | Varied | <21 | 11067 | Consumer Assessment of Health Providers  and Systems (CAHPS) surveys | Proxy | Telephone | Good |
| **59** | Fustino, Nicholas J; Moore, Paige; Viers, Sandy; Cheyne, Ken, 2019, USA[^59^](https://www.zotero.org/google-docs/?5maflr) | To increase patient experience care provider scores and assess how improvement efforts impact practice expansion, patient complaint rates, and staff engagement. | Prospective interventional study | 5 years | General | Multiclinic children’s hospital (tertiary care, ambulatory practices) | NR | 11705 | Press-Ganey Satisfaction Survey | Proxy | Paper and electronic | Fair |
| **60** | Fustino, Nicholas J; Kochanski, Justin J, 2015, USA[^60^](https://www.zotero.org/google-docs/?Pr21PL) | To improve patient satisfaction with care in an outpatient clinic. | Quality improvement initiative | 2 years | Hematology- oncology disorders | Outpatient clinic | <18 | 207 | Press-Ganey Satisfaction Survey | Proxy | Paper | Fair |
| **61** | Davis-Dao, Carol A; Ehwerhemuepha, Louis; Chamberlin, Joshua D; Feaster, William; Khoury, Antoine E; Fortier, Michelle A; Kain, Zeev N, 2020, USA[^61^](https://www.zotero.org/google-docs/?8YSiyz) | To compile a list of factors that predict patient satisfaction in a pediatric urology setting. | Cross- sectional | 2 years | Urologic disorders | Urology outpatient clinic | NR | 3232 | NRC Health Patient Survey | Proxy | Telephone and electronic | Good |
| **62** | Fustino, Nicholas J; Wohlfeil, Margo; Smith, Hayden L, 2018, USA[^62^](https://www.zotero.org/google-docs/?00upFd) | To determine key drivers of high patient satisfaction and factors which increase the probability of the patient recommending the practice to others. | Cross- sectional descriptive survey | 2 years 5 months | Hematology- oncology disorder | Hematology-oncology unit | <21 | 281 | Consumer Assessment of Healthcare Providers and Systems Clinician &  Group Survey (CG-CAHPS) | NR | Electronic | Fair |
| **63** | Furness, Caroline L; Smith, Lesley; Morris, Eva; Brocklehurst, Caroline; Daly, Sasha; Hough, Rachael E, 2017, UK[^63^](https://www.zotero.org/google-docs/?Nh3ON3) | To identify areas of improvement in teen and young adult cancer care and to see if there has been a stepwise increase in patient satisfaction over time. Research also assessed whether care in a principle treatment centre affects patient experience. | Retrospective cohort | 4 years | Cancer | Varied | 13-24 | 1367 | The national cancer patient experience survey | Patient | Paper | Good |
| **64** | Gray, J E; Safran, C; Davis, R B; Pompilio-Weitzner, G; Stewart, J E; Zaccagnini, L; Pursley, D, 2000, USA[^64^](https://www.zotero.org/google-docs/?dgVrPh) | To evaluate the efficacy of an Internet-based telemedicine program in the NICU through assessing parental quality assessments of their care. | Randomized control trial | Nov 1997- April 1999 | General (NICU - low birth weight) | NICU | Neonates | 31 | The Picker Institute’s Neonatal Intensive Care Unit FamilySatisfaction survey | Proxy | NR | Good |
| **65** | Norman, Shelley Marie; Ford, Tamsin; Henley, William; Goodman, Robert, 2016, UK[^65^](https://www.zotero.org/google-docs/?RiuwDd) | To examine the associations between patient-reported outcome measures, practitioner- reported outcome measures, and PREMs. | Cohort study | 1 year | Mental health | CAMHS facilities | <16 | 189 | Experiences of Services Questionnaire | Proxy | NR | Good |
| **66** | Madan, Alok; Sharp, Carla; Newlin, Elizabeth; Vanwoerden, Salome; Fowler, J Christopher, 2016, USA[^66^](https://www.zotero.org/google-docs/?7ir5lM) | To assess patient and parental satisfaction with inpatient psychiatric care and to examine the association between satisfaction of patient outcomes. | Cohort study | 1 year 9 months | Psychiatric disorders | Inpatient care unit | Adolescents | 129 | McLean Hospital’s Perception of Care survey | Both the patient and proxy | NR | Good |
| **67** | Mah, Jean K; Tough, Suzanne; Fung, Thomas; Douglas-England, Kathleen; Verhoef, Marja, 2006, Canada[^67^](https://www.zotero.org/google-docs/?W0v4Io) | To collect patient responses to a PREM covering patient satisfaction and FCC, and to see how the responses related to the patients’ HRQoL. Additionally, patient responses were compared to their parents to identify possible discrepancies. | Cross- sectional | 4 months | Neurologic diseases | Ambulatory clinic | 12-18 | 104 | MPOC-20 ; Give Youth a Voice survey (GYV) | MPOC-20 - proxy; GYV - patient | NR | Good |
| **68** | Hummel, Kevin; Presson, Angela P; Millar, Morgan M; Larsen, Gitte; Kadish, Howard; Olson, Lenora M, 2020, USA[^68^](https://www.zotero.org/google-docs/?Pv5ymT) | To determine key factors for family satisfaction in the PICU and to formulate the best approach for survey dissemination. | Mixed methods | 7 months | General | PICU | <18 | 206 | Pediatric Family Satisfaction-ICU (pFS-ICU) | Proxy | Electronic | Good |
| **69** | Toomey, Sara L; Elliott, Marc N; Zaslavsky, Alan M; Quinn, Jessica; Klein, David J; Wagner, Stephanie; Thomson, Cassandra; Wu, Melody; Onorato, Sarah; Schuster, Mark A, 2019, USA[^69^](https://www.zotero.org/google-docs/?WujSJA) | To assess whether the administration of discharge surveys with tablets increases patient responsiveness and representativeness. | Quasi-experimental | 4 months | General | Tertiary inpatient care unit | <18 | 1184 | Child HCAHPS | Proxy | Paper and electronic | Good |
| **70** | Uhl, Tammy; Fisher, Kimberley; Docherty, Sharron L; Brandon, Debra H, 2013, USA[^70^](https://www.zotero.org/google-docs/?Tmd00s) | To improve PCC and FCC through the analysis of parent-completed PREMs. | Mixed methods | 5 months | Chronic diseases | Tertiary inpatient care unit | <18 | 134 | The Children's Hospital Boston Inpatient Experience Survey | Proxy | Paper and electronic | Fair |
| **71** | Viner RM, 2007, UK[^71^](https://www.zotero.org/google-docs/?wOTjHd) | To evaluate the effects of creating an adolescent-specific ward on perceived care quality, relative to treating these patients in child or adult settings. | Secondary data analysis | NR | General | Varied | <17 | 59815 | National English Young Patient Survey 2004 (YPS) | Both the patient and proxy | Paper | Good |
| **72** | Christensen, Anna L; Brown, Jonathan D; Wissow, Lawrence S; Cook, Benjamin, 2016, USA[^72^](https://www.zotero.org/google-docs/?JemO07) | To assess parental satisfaction with the PCC and FCC demonstrated by medical assistants, and to compare these survey results with other elements of their care. | Secondary data analysis | NR | General | Primary clinical care | 18months -16 | 360 | Consultation and Relational Empathy measure (CARE) | Proxy | NR | Good |
| **73** | Barsoom R.R.; Maugans T.A.; Burrows J.F.; Rosen P., 2017, USA[^73^](https://www.zotero.org/google-docs/?9eqdgB) | To perform clinical quality improvements through the dissemination of PREMs after medical visits. | Cross- sectional | 3 years | Neurologic diseases | Outpatient care | NR | 458 | Press Ganey Satisfaction Survey | Proxy | NR | Good |
| **74** | Barber AJ; Tischler VA; Healy E, 2006, USA[^74^](https://www.zotero.org/google-docs/?ayUEDy) | To evaluate the likeness of CAMHS PREMs that were completed by either themselves or their carer. | Cross- sectional | NR | Mental health | Outpatient care | ≥4 | 118 | Experience of Service Questionnaire (ESQ) | Both the patient and the proxy | Paper | Good |
| **75** | Ahmed, Sarah; Miller, Jonathan; Burrows, James F; Bertha, Ben Khallouq; Rosen, Paul, 2017, USA[^75^](https://www.zotero.org/google-docs/?zq3QKR) | To determine the primary factors that are predictive of positive patient experience. | Retrospective | 3 years | Dermatological diseases | Tertiary outpatient care | NR | 516 | Press Ganey Satisfaction Survey | Proxy | Paper and electronic | Good |
| **76** | Chen, Alex Y; Elliott, Marc N; Spritzer, Karen L; Brown, Julie A; Skootsky, Samuel A; Rowley, Cliff; Hays, Ron D, 2012, USA[^76^](https://www.zotero.org/google-docs/?jXsA6Z) | To assess and contrast the experiences of pediatric and adult patients. | Observational study | 2 years | General | Primary clinical care | NR | 668 parents/ 7823 adults | Consumer Assessment of Healthcare Providers and Systems Clinician & Group 1.0 (CG-CAHPS 1.0) | Proxy | Paper and electronic | Good |
| **77** | Burnet, Deborah; Gunter, Kathryn E; Nocon, Robert S; Gao, Yue; Jin, Janel; Fairchild, Paige; Chin, Marshall H, 2014, USA[^77^](https://www.zotero.org/google-docs/?WjW6FG) | To evaluate the effects of increasing PCC characteristics on patient perceptions of their quality of care. | Cross- sectional | 1 year 6 months | General | Primary clinical care | 1-17 | 440 | Consumer Assessment of Healthcare Providers and Systems Clinician & Group (CAHPS-CG) | Proxy | Paper | Good |
| **78** | Brousseau, David C; Mukonje, Terence; Brandow, Amanda M; Nimmer, Mark; Panepinto, Julie A, 2009, USA[^78^](https://www.zotero.org/google-docs/?G2LKHh) | To compare parental dissatisfaction with sickle cell care to the dissatisfaction of parents receiving asthma or general pediatric care. | Cross- sectional | 3 years | Sickle cell diseases, asthma, general pediatrics | Inpatient care unit | 2-18 | 639 | Picker Inpatient Survey | Proxy | NR | Good |
| **79** | Bumpers, Bernadette; Dearmon, Valorie; Dycus, Paula, 2019, USA[^79^](https://www.zotero.org/google-docs/?zhETdm) | To examine whether the implementation of a “communication bundle” improves parental experiences with patient-nurse communication. | Quality improvement | 15 weeks | Orthopedic and hematologic diseases | Tertiary inpatient care unit | NR | NR | Child HCAHPS | Proxy | Telephone | Fair |
| **80** | Boss, Emily F; Thompson, Richard E, 2012, USA[^80^](https://www.zotero.org/google-docs/?NH3rm9) | To assess the satisfaction ratings of otolaryngic outpatients and whether these ratings differ between age groups. | Cross- sectional | 1 year | Otolaryngologic diseases | Outpatient care | <17 | 5996 | Press Ganey Medical Practice surveys | NR | NR | Good |
| **81** | Boss, Emily F; Thompson, Richard E, 2013, USA[^81^](https://www.zotero.org/google-docs/?VlU34o) | To examine how a teaching versus non-teaching setting influences parental satisfaction with otolaryngic care. | Cross- sectional | 1 year | Otolaryngologic diseases | Outpatient care | <17 | 4704 | Press Ganey Medical Practice surveys | Proxy | NR | Good |
| **82** | Bal, Chandan; AlNajjar, Mohammad; Thull-Freedman, Jennifer; Pols, Erin; McFetridge, Ashley; Stang, Antonia S, 2020, Canada[^82^](https://www.zotero.org/google-docs/?VUDfYu) | To collect information on patient experiences in an emergency department and to assess how care perceptions may differ between a child and their parent(s). Research also investigated whether extrinsic factors may be impacting survey responses. | Cross- sectional | 2 months | Emergency department | Tertiary clinical care | <17 | 346 | The patient- reported experience measure (PREM) for children in urgent and emergency care. | Both the patient and proxy | Paper and electronic | Good |
| **83** | Allam, Shalini D; Mehta, Mary; Ben Khallouq, Bertha; Burrows, James F; Rosen, Paul, 2017, USA[^83^](https://www.zotero.org/google-docs/?9RLuRy) | To identify components of patient care that are indicative of high patient satisfaction in cardiology settings. | Retrospective cohort | 2 years 2 months | Cardiovascular diseases | Outpatient care | NR | 2468 | Press Ganey Physician Specialties Survey | Proxy | Paper and electronic | Good |

References:

[1. Krugman SD, Suggs A, Photowala HY, Beck A. Redefining the Community Pediatric Hospitalist: The Combined Pediatric ED/Inpatient Unit. *Pediatr Emerg Care*. 2007;23(1):33-37. doi:10.1097/01.pec.0000248685.94647.01](https://www.zotero.org/google-docs/?WAbUV2)

[2. Matziou V, Boutopoulou B, Chrysostomou A, Vlachioti E, Mantziou T, Petsios K. Parents’ satisfaction concerning their child’s hospital care. *Jpn J Nurs Sci*. 2011;8(2):163-173. doi:https://doi.org/10.1111/j.1742-7924.2010.00171.x](https://www.zotero.org/google-docs/?WAbUV2)

[3. Williams F, McCafferty A, Dunkley C, Kirkpatrick M. A UK survey of the experience of service provision for children and young people with epilepsy. *Seizure*. 2018;60:80-85. doi:10.1016/j.seizure.2018.06.007](https://www.zotero.org/google-docs/?WAbUV2)

[4. Voos KC, Ross G, Ward MJ, Yohay A-L, Osorio SN, Perlman JM. Effects of implementing family-centered rounds (FCRs) in a neonatal intensive care unit (NICU). *J Matern Fetal Neonatal Med*. 2011;24(11):1403-1406. doi:10.3109/14767058.2011.596960](https://www.zotero.org/google-docs/?WAbUV2)

[5. Nichol JR, Fu R, French K, Momberger J, Handel DA. Association Between Patient and Emergency Department Operational Characteristics and Patient Satisfaction Scores in a Pediatric Population. *Pediatr Emerg Care*. 2016;32(3):139-141. doi:10.1097/PEC.0000000000000723](https://www.zotero.org/google-docs/?WAbUV2)

[6. Sigurdardottir AO, Garwick AW, Svavarsdottir EK. The importance of family support in pediatrics and its impact on healthcare satisfaction. *Scand J Caring Sci*. 2017;31(2):241-252. doi:https://doi.org/10.1111/scs.12336](https://www.zotero.org/google-docs/?WAbUV2)

[7. Timmer A, Peplies J, Westphal M, et al. Transition from pediatric to adult medical care - A survey in young persons with inflammatory bowel disease. *PLoS ONE*. 2017;12(5):e0177757.](https://www.zotero.org/google-docs/?WAbUV2)

[8. Sleath B, Ayala GX, Washington D, et al. Caregiver rating of provider participatory decision-making style and caregiver and child satisfaction with pediatric asthma visits. *Patient Educ Couns*. 2011;85(2):286-289. doi:10.1016/j.pec.2010.09.016](https://www.zotero.org/google-docs/?WAbUV2)

[9. de Wit M, de Waal HAD, Bokma JA, et al. Monitoring and discussing health-related quality of life in adolescents with type 1 diabetes improve psychosocial well-being: a randomized controlled trial. *Diabetes Care*. 2008;31(8):1521+.](https://www.zotero.org/google-docs/?WAbUV2)

[10. Cohen E, Austin J, Weinstein M, Matlow A, Redelmeier DA. Care of Children Isolated for Infection Control: A Prospective Observational Cohort Study. *Pediatrics*. 2008;122(2):e411-e415. doi:10.1542/peds.2008-0181](https://www.zotero.org/google-docs/?WAbUV2)

[11. Galbraith AA, Semura J, McAninch-Dake B, Anderson N, Christakis DA. Emergency Department Use and Perceived Delay in Accessing Illness Care Among Children With Medicaid. *Ambul Pediatr*. 2004;4(6):509-513. doi:10.1367/A04-008R.1](https://www.zotero.org/google-docs/?WAbUV2)

[12. Adams EK, Ketsche P, Zhou M, Minyard K. Access and satisfaction among children in Georgia’s Medicaid program and SCHIP: 2000 to 2003. *Health Care Financ Rev*. 2008;29(3):43+.](https://www.zotero.org/google-docs/?WAbUV2)

[13. Tuñas MC, Varela F, Rama E, et al. Cuidados intensivos pediátricos: calidad percibida por los progenitores. *Metas Enferm*. 2018;21. doi:10.35667/MetasEnf.2019.21.1003081208](https://www.zotero.org/google-docs/?WAbUV2)

[14. Mah JK, Tough S, Fung T, Douglas-england K, Verhoef M. Parents’ Global Rating of Mental Health Correlates with SF-36 Scores and Health Services Satisfaction. *Qual Life Res*. 2006;15(8):1395-1401. doi:http://dx.doi.org.proxy.queensu.ca/10.1007/s11136-006-0014-z](https://www.zotero.org/google-docs/?WAbUV2)

[15. Mason SL, Chike-Harris KE, Gyr BM, Johnson E. Effect of Facilitated Discharge in Pediatric Orthopedic Patients at an Academic Medical Facility. *J Pediatr Health Care*. 2019;33(1):58-63. doi:10.1016/j.pedhc.2018.06.003](https://www.zotero.org/google-docs/?WAbUV2)

[16. Stefánsdóttir S, Thóra Egilson S. Diverging perspectives on children’s rehabilitation services: a mixed-methods study. *Scand J Occup Ther*. 2016;23(5):374-382. doi:10.3109/11038128.2015.1105292](https://www.zotero.org/google-docs/?WAbUV2)

[17. Siebes RC, Wijnroks L, Ketelaar M, van Schie PEM, Gorter JW, Vermeer A. Parent participation in paediatric rehabilitation treatment centres in the Netherlands: a parents’ viewpoint. *Child Care Health Dev*. 2007;33(2):196-205. doi:10.1111/j.1365-2214.2006.00636.x](https://www.zotero.org/google-docs/?WAbUV2)

[18. Van Riper M. Family-provider relationships and well-being in families with preterm infants in the NICU. *Heart Lung J Crit Care*. 2001;30(1):74-84. doi:10.1067/mhl.2001.110625](https://www.zotero.org/google-docs/?WAbUV2)

[19. Nieman CL, Benke JR, Ishman SL, Smith DF, Boss EF. Whose Experience Is Measured?: A Pilot Study of Patient Satisfaction Demographics in Pediatric Otolaryngology. *The Laryngoscope*. 2014;124(1):290-294. doi:10.1002/lary.24307](https://www.zotero.org/google-docs/?WAbUV2)

[20. Wells RD, Dahl B, Wilson SD. Pediatric hospitalists: quality care for the underserved? *Am J Med Qual Off J Am Coll Med Qual*. 2001;16(5):174-180. doi:10.1177/106286060101600505](https://www.zotero.org/google-docs/?WAbUV2)

[21. Toomey SL, Elliott MN, Zaslavsky AM, et al. Variation in Family Experience of Pediatric Inpatient Care As Measured by Child HCAHPS. *Pediatrics*. 2017;139(4). doi:10.1542/peds.2016-3372](https://www.zotero.org/google-docs/?WAbUV2)

[22. Terwiel M, Alsem MW, Siebes RC, Bieleman K, Verhoef M, Ketelaar M. Family-centred service: differences in what parents of children with cerebral palsy rate important. *Child Care Health Dev*. 2017;43(5):663-669. doi:10.1111/cch.12460](https://www.zotero.org/google-docs/?WAbUV2)

[23. Williams G, Pattison G, Mariathas C, Lazar J, Rashied M. Improving parental satisfaction in pediatric orthopaedics. *J Pediatr Orthop*. 2011;31(5):610-615. doi:10.1097/BPO.0b013e3182203955](https://www.zotero.org/google-docs/?WAbUV2)

[24. Nieman CL, Benke JR, Boss EF. Does Race/Ethnicity or Socioeconomic Status Influence Patient Satisfaction in Pediatric Surgical Care? *Otolaryngol--Head Neck Surg Off J Am Acad Otolaryngol-Head Neck Surg*. 2015;153(4):620-628. doi:10.1177/0194599815590592](https://www.zotero.org/google-docs/?WAbUV2)

[25. Tothy AS, Limper HM, Driscoll J, Bittick N, Howell MD. The Ask Me to Explain Campaign: A 90-Day Intervention to Promote Patient and Family Involvement in Care in a Pediatric Emergency Department. *Jt Comm J Qual Patient Saf*. 2016;42(6):281-AP1. doi:10.1016/S1553-7250(16)42037-4](https://www.zotero.org/google-docs/?WAbUV2)

[26. Tilly-Gratton A, Nadon MA, Houle A, Pelaez S, Ducharme FM. What convinces parents of children with asthma to adhere to maintenance inhaled corticosteroids? *Can J Respir Crit Care Sleep Med*. 2018;2(3):147-154. doi:10.1080/24745332.2018.1450101](https://www.zotero.org/google-docs/?WAbUV2)

[27. Sonneveld HM, Strating MMH, Staa AL van, Nieboer AP. Gaps in transitional care: what are the perceptions of adolescents, parents and providers? *Child Care Health Dev*. 2013;39(1):69-80. doi:https://doi.org/10.1111/j.1365-2214.2011.01354.x](https://www.zotero.org/google-docs/?WAbUV2)

[28. Siebes RC, Wijnroks L, Ketelaar M, van Schie PEM, Vermeer A, Gorter JW. One-year stability of the Measure of Processes of Care. *Child Care Health Dev*. 2007;33(5):604-610. doi:10.1111/j.1365-2214.2007.00726.x](https://www.zotero.org/google-docs/?WAbUV2)

[29. Locke R, Stefano M, Koster A, Taylor B, Greenspan J. Optimizing patient/caregiver satisfaction through quality of communication in the pediatric emergency department. *Pediatr Emerg Care*. 2011;27(11):1016-1021. doi:10.1097/PEC.0b013e318235be06](https://www.zotero.org/google-docs/?WAbUV2)

[30. Camilon PR, Levi JR, Carrion RA, Josephson GD. Physician impact on the patient and family experience in a pediatric otolaryngology practice. *The Laryngoscope*. 2019;129(11):2610-2613. doi:https://doi.org/10.1002/lary.27596](https://www.zotero.org/google-docs/?WAbUV2)

[31. Shaw KL, Southwood TR, McDonagh JE, British Society of Paediatric and Adolescent Rheumatology. Young people’s satisfaction of transitional care in adolescent rheumatology in the UK. *Child Care Health Dev*. 2007;33(4):368-379. doi:10.1111/j.1365-2214.2006.00698.x](https://www.zotero.org/google-docs/?WAbUV2)

[32. Shaw KL, Watanabe A, Rankin E, McDonagh JE. Walking the talk. Implementation of transitional care guidance in a UK paediatric and a neighbouring adult facility. *Child Care Health Dev*. 2014;40(5):663-670. doi:10.1111/cch.12110](https://www.zotero.org/google-docs/?WAbUV2)

[33. Shevell M, Oskoui M, Wood E, et al. Family-centred health care for children with cerebral palsy. *Dev Med Child Neurol*. 2019;61(1):62-68. doi:10.1111/dmcn.14053](https://www.zotero.org/google-docs/?WAbUV2)

[34. Singh SC, Sheth RD, Burrows JF, Rosen P. Factors Influencing Patient Experience in Pediatric Neurology. *Pediatr Neurol*. 2016;60:37-41. doi:10.1016/j.pediatrneurol.2016.04.002](https://www.zotero.org/google-docs/?WAbUV2)

[35. Singleton IM, Garfinkel RJ, Malone JB, Temkit MH, Belthur MV. Determinants of caregiver satisfaction in pediatric orthopedics. *J Pediatr Orthop Part B*. 2021;30(4):393-398. doi:10.1097/BPB.0000000000000778](https://www.zotero.org/google-docs/?WAbUV2)

[36. Sng QW, Kirk AHP, Buang SNH, Lee JH. The Impact of Ethnic and Cultural Differences on Parental Satisfaction in the PICU. *Pediatr Crit Care Med J Soc Crit Care Med World Fed Pediatr Intensive Crit Care Soc*. 2017;18(4):e167-e175. doi:10.1097/PCC.0000000000001095](https://www.zotero.org/google-docs/?WAbUV2)

[37. Schreiber J, Benger J, Salls J, Marchetti G, Reed L. Parent perspectives on rehabilitation services for their children with disabilities: a mixed methods approach. *Phys Occup Ther Pediatr*. 2011;31(3):225-238. doi:10.3109/01942638.2011.565865](https://www.zotero.org/google-docs/?WAbUV2)

[38. Rahi JS, Manaras I, Tuomainen H, Hundt GL. Health services experiences of parents of recently diagnosed visually impaired children. *Br J Ophthalmol*. 2005;89(2):213-218. doi:10.1136/bjo.2004.051409](https://www.zotero.org/google-docs/?WAbUV2)

[39. Rahi JS, Manaras I, Tuomainen H, Hundt GL. Meeting the needs of parents around the time of diagnosis of disability among their children: evaluation of a novel program for information, support, and liaison by key workers. *Pediatrics*. 2004;114(4):e477-482. doi:10.1542/peds.2004-0240](https://www.zotero.org/google-docs/?WAbUV2)

[40. Hargreaves DS, McDonagh JE, Viner RM. Validation of You’re Welcome Quality Criteria for Adolescent Health Services Using Data From National Inpatient Surveys in England. *J Adolesc Health*. 2013;52(1):50-57.e1. doi:10.1016/j.jadohealth.2012.04.005](https://www.zotero.org/google-docs/?WAbUV2)

[41. Hargreaves DS, Sizmur S, Pitchforth J, et al. Children and young people’s versus parents’ responses in an English national inpatient survey. *Arch Dis Child*. 2018;103(5):486-491. doi:10.1136/archdischild-2017-313801](https://www.zotero.org/google-docs/?WAbUV2)

[42. Myrhaug HT, Jahnsen R, Østensjø S. Family-centred practices in the provision of interventions and services in primary health care: A survey of parents of preschool children with cerebral palsy. *J Child Health Care Prof Work Child Hosp Community*. 2016;20(1):109-119. doi:10.1177/1367493514551312](https://www.zotero.org/google-docs/?WAbUV2)

[43. Nagarajan N, Rahman S, Boss EF. Are There Racial Disparities in Family-Reported Experiences of Care in Inpatient Pediatrics? *Clin Pediatr (Phila)*. 2017;56(7):619-626. doi:10.1177/0009922816668497](https://www.zotero.org/google-docs/?WAbUV2)

[44. Peng FB, Burrows JF, Shirley ED, Rosen P. Unlocking the Doors to Patient Satisfaction in Pediatric Orthopaedics. *J Pediatr Orthop*. 2018;38(8):398-402. doi:10.1097/BPO.0000000000000837](https://www.zotero.org/google-docs/?WAbUV2)

[45. Petitgout JM. Implementation and Evaluation of a Unit-Based Discharge Coordinator to Improve the Patient Discharge Experience. *J Pediatr Health Care Off Publ Natl Assoc Pediatr Nurse Assoc Pract*. 2015;29(6):509-517. doi:10.1016/j.pedhc.2015.02.004](https://www.zotero.org/google-docs/?WAbUV2)

[46. Kemp KA, Ahmed S, Quan H, Johnson D, Santana MJ. Family Experiences of Pediatric Inpatient Care in Alberta, Canada: Results From the Child HCAHPS Survey. *Hosp Pediatr*. 2018;8(6):338-344. doi:10.1542/hpeds.2017-0191](https://www.zotero.org/google-docs/?WAbUV2)

[47. Kleinsorge CA, Roberts MC, Roy KM, Rapoff MA. The program evaluation of services in a primary care clinic: attaining a medical home. *Clin Pediatr (Phila)*. 2010;49(6):548-559. doi:10.1177/0009922809358615](https://www.zotero.org/google-docs/?WAbUV2)

[48. Knapp C, Madden V, Sloyer P, Shenkman E. Effects of an Integrated Care System on quality of care and satisfaction for children with special health care needs. *Matern Child Health J*. 2012;16(3):579-586. doi:10.1007/s10995-011-0778-9](https://www.zotero.org/google-docs/?WAbUV2)

[49. Knox V, Menzies S. Using the Measure of Processes of Care to Assess Parents’ Views of a Paediatric Therapy Service. *Br J Occup Ther*. 2005;68(3):110-116. doi:10.1177/030802260506800303](https://www.zotero.org/google-docs/?WAbUV2)

[50. Kuo DZ, Sisterhen LL, Sigrest TE, Biazo JM, Aitken ME, Smith CE. Family Experiences and Pediatric Health Services Use Associated With Family-Centered Rounds. *Pediatrics*. 2012;130(2):299-305. doi:10.1542/peds.2011-2623](https://www.zotero.org/google-docs/?WAbUV2)

[51. Koves IH, Boucher A, Ismail D, Donath S, Cameron FJ. Satisfaction of care in a tertiary level diabetes clinic: correlations with diabetes knowledge, clinical outcome and health-related quality of life. *J Paediatr Child Health*. 2008;44(7-8):432-437. doi:10.1111/j.1440-1754.2008.01317.x](https://www.zotero.org/google-docs/?WAbUV2)

[52. Miceli PJ, Clark PA. Your patient--my child: seven priorities for improving pediatric care from the parent’s perspective. *J Nurs Care Qual*. 2005;20(1):43-53; quiz 54-55. doi:10.1097/00001786-200501000-00008](https://www.zotero.org/google-docs/?WAbUV2)

[53. Janhunen K, Kankkunen P, Kvist T. Quality of Pediatric Emergency Care as Assessed by Children and Their Parents. *J Nurs Care Qual*. 2019;34(2):180-184. doi:10.1097/NCQ.0000000000000346](https://www.zotero.org/google-docs/?WAbUV2)

[54. Lee B, Hollenbeck-Pringle D, Goldman V, Biondi E, Alverson B. Are Caregivers Who Respond to the Child HCAHPS Survey Reflective of All Hospitalized Pediatric Patients? *Hosp Pediatr*. 2019;9(3):162-169. doi:10.1542/hpeds.2018-0139](https://www.zotero.org/google-docs/?WAbUV2)

[55. Hurtubise K, Shanks R, Benard L. The Design, Implementation, and Evaluation of a Physiotherapist-Led Clinic for Orthopedic Surveillance for Children with Cerebral Palsy. *Phys Occup Ther Pediatr*. 2017;37(4):399-413. doi:10.1080/01942638.2017.1280869](https://www.zotero.org/google-docs/?WAbUV2)

[56. Harder VS, Krulewitz J, Jones C, Wasserman RC, Shaw JS. Effects of Patient-centered Medical Home Transformation on Child Patient Experience. *J Am Board Fam Med JABFM*. 2016;29(1):60-68. doi:10.3122/jabfm.2016.01.150066](https://www.zotero.org/google-docs/?WAbUV2)

[57. Groleger Sršen K, Vidmar G, Sočan G, Zupan A. Parental evaluation of processes of care in relation to the child, parent and family characteristics. *Int J Rehabil Res Int Z Rehabil Rev Int Rech Readaptation*. 2014;37(3):220-228. doi:10.1097/MRR.0000000000000068](https://www.zotero.org/google-docs/?WAbUV2)

[58. Hall AG, Landry AY, Lemak CH, Boyle EL, Duncan RP. Reported experiences with Medicaid managed care models among parents of children. *Matern Child Health J*. 2014;18(3):544-553. doi:10.1007/s10995-013-1270-5](https://www.zotero.org/google-docs/?WAbUV2)

[59. Fustino NJ, Moore P, Viers S, Cheyne K. Improving Patient Experience of Care Providers in a Multispecialty Ambulatory Pediatrics Practice. *Clin Pediatr (Phila)*. 2019;58(1):50-59. doi:10.1177/0009922818806309](https://www.zotero.org/google-docs/?WAbUV2)

[60. Fustino NJ, Kochanski JJ. Improving Patient Satisfaction in a Midsize Pediatric Hematology-Oncology Outpatient Clinic. *J Oncol Pract*. 2015;11(5):416-420. doi:10.1200/JOP.2015.004911](https://www.zotero.org/google-docs/?WAbUV2)

[61. Davis-Dao CA, Ehwerhemuepha L, Chamberlin JD, et al. Keys to improving patient satisfaction in the pediatric urology clinic: A starting point. *J Pediatr Urol*. 2020;16(3):377-383. doi:10.1016/j.jpurol.2020.03.013](https://www.zotero.org/google-docs/?WAbUV2)

[62. Fustino NJ, Wohlfeil M, Smith HL. Determination of Key Drivers of Patient Experience in a Midsize Pediatric Hematology-Oncology Ambulatory Clinic. *Ochsner J*. 2018;18(4):332-338. doi:10.31486/toj.18.0091](https://www.zotero.org/google-docs/?WAbUV2)

[63. Furness CL, Smith L, Morris E, Brocklehurst C, Daly S, Hough RE. Cancer Patient Experience in the Teenage Young Adult Population- Key Issues and Trends Over Time: An Analysis of the United Kingdom National Cancer Patient Experience Surveys 2010-2014. *J Adolesc Young Adult Oncol*. 2017;6(3):450-458. doi:10.1089/jayao.2016.0058](https://www.zotero.org/google-docs/?WAbUV2)

[64. Gray JE, Safran C, Davis RB, et al. Baby CareLink: using the internet and telemedicine to improve care for high-risk infants. *Pediatrics*. 2000;106(6):1318-1324. doi:10.1542/peds.106.6.1318](https://www.zotero.org/google-docs/?WAbUV2)

[65. Norman S, Ford T, Henley W, Goodman R. A comparison of parent reported outcome with experience of services. *J Child Serv*. 2016;11:157-169. doi:10.1108/JCS-04-2015-0015](https://www.zotero.org/google-docs/?WAbUV2)

[66. Madan A, Sharp C, Newlin E, Vanwoerden S, Fowler JC. Adolescents Are Less Satisfied with Inpatient Psychiatric Care than Their Parents: Does It Matter? *J Healthc Qual*. n/a(n/a). doi:https://doi.org/10.1111/jhq.12081](https://www.zotero.org/google-docs/?WAbUV2)

[67. Mah JK, Tough S, Fung T, Douglas-England K, Verhoef M. Adolescent quality of life and satisfaction with care. *J Adolesc Health Off Publ Soc Adolesc Med*. 2006;38(5):607.e1-7. doi:10.1016/j.jadohealth.2005.08.004](https://www.zotero.org/google-docs/?WAbUV2)

[68. Hummel K, Presson AP, Millar MM, Larsen G, Kadish H, Olson LM. An Assessment of Clinical and System Drivers of Family Satisfaction in the PICU. *Pediatr Crit Care Med J Soc Crit Care Med World Fed Pediatr Intensive Crit Care Soc*. 2020;21(10):e888-e897. doi:10.1097/PCC.0000000000002394](https://www.zotero.org/google-docs/?WAbUV2)

[69. Toomey SL, Elliott MN, Zaslavsky AM, et al. Improving Response Rates and Representation of Hard-to-Reach Groups in Family Experience Surveys. *Acad Pediatr*. 2019;19(4):446-453. doi:10.1016/j.acap.2018.07.007](https://www.zotero.org/google-docs/?WAbUV2)

[70. Uhl T, Fisher K, Docherty SL, Brandon DH. Insights into patient and family-centered care through the hospital experiences of parents. *J Obstet Gynecol Neonatal Nurs JOGNN*. 2013;42(1):121-131. doi:10.1111/1552-6909.12001](https://www.zotero.org/google-docs/?WAbUV2)

[71. Viner RM. Do Adolescent Inpatient Wards Make a Difference? Findings From a National Young Patient Survey. *Pediatrics*. 2007;120(4):749-755. doi:10.1542/peds.2006-3293](https://www.zotero.org/google-docs/?WAbUV2)

[72. Christensen AL, Brown JD, Wissow LS, Cook B. Spillover of Ratings of Patient- and Family-Centered Care. *J Ambulatory Care Manage*. 2016;39(4):308-315. doi:10.1097/JAC.0000000000000133](https://www.zotero.org/google-docs/?WAbUV2)

[73. Barsoom RR, Maugans TA, Burrows JF, Rosen P. Exploring patient and family satisfaction in pediatric neurological surgery. *Interdiscip Neurosurg*. 2017;7:1-3. doi:10.1016/j.inat.2016.10.002](https://www.zotero.org/google-docs/?WAbUV2)

[74. Barber AJ, Tischler VA, Healy E. Consumer satisfaction and child behaviour problems in child and adolescent mental health services. *J Child Health Care Prof Work Child Hosp Community*. 2006;10(1):9-21. doi:10.1177/1367493506060200](https://www.zotero.org/google-docs/?WAbUV2)

[75. Ahmed S, Miller J, Burrows JF, Bertha BK, Rosen P. Evaluation of patient satisfaction in pediatric dermatology. *Pediatr Dermatol*. 2017;34(6):668-672. doi:10.1111/pde.13294](https://www.zotero.org/google-docs/?WAbUV2)

[76. Chen AY, Elliott MN, Spritzer KL, et al. Differences in CAHPS reports and ratings of health care provided to adults and children. *Med Care*. 2012;50 Suppl:S35-39. doi:10.1097/MLR.0b013e3182610a88](https://www.zotero.org/google-docs/?WAbUV2)

[77. Burnet D, Gunter KE, Nocon RS, et al. Medical Home Characteristics and the Pediatric Patient Experience. *Med Care*. 2014;52:S56. doi:10.1097/MLR.0000000000000238](https://www.zotero.org/google-docs/?WAbUV2)

[78. Brousseau DC, Mukonje T, Brandow AM, Nimmer M, Panepinto JA. Dissatisfaction with hospital care for children with sickle cell disease not due only to race and chronic disease. *Pediatr Blood Cancer*. 2009;53(2):174-178. doi:10.1002/pbc.22039](https://www.zotero.org/google-docs/?WAbUV2)

[79. Bumpers B, Dearmon V, Dycus P. Impacting the Patient’s Experience in a Children’s Hospital Using a Communication Bundle Strategy. *J Nurs Care Qual*. 2019;34(1):86-90. doi:10.1097/NCQ.0000000000000336](https://www.zotero.org/google-docs/?WAbUV2)

[80. Boss EF, Thompson RE. Patient experience in outpatient pediatric otolaryngology. *The Laryngoscope*. 2012;122(10):2304-2310. doi:https://doi.org/10.1002/lary.23364](https://www.zotero.org/google-docs/?WAbUV2)

[81. Boss EF, Thompson RE. Patient experience in the pediatric otolaryngology clinic: Does the teaching setting influence parent satisfaction? *Int J Pediatr Otorhinolaryngol*. 2013;77(1):59-64. doi:10.1016/j.ijporl.2012.09.030](https://www.zotero.org/google-docs/?WAbUV2)

[82. Bal C, AlNajjar M, Thull-Freedman J, Pols E, McFetridge A, Stang AS. Patient Reported Experience in a Pediatric Emergency Department. *J Patient Exp*. 2020;7(1):116-123. doi:10.1177/2374373519826560](https://www.zotero.org/google-docs/?WAbUV2)

[83. Allam SD, Mehta M, Ben Khallouq B, Burrows JF, Rosen P. Key drivers of patient experience in ambulatory paediatric cardiology. *Cardiol Young*. 2017;27(8):1585-1590. doi:10.1017/S1047951117000841](https://www.zotero.org/google-docs/?WAbUV2)
