# Supplementary material for: Use of Patient-Reported Experience Measures in Pediatric Care: A Systematic Review
Source: Front Pediatr. 2021 Dec 20;9:753536. doi: 10.3389/fped.2021.753536 (PMC8721567; doi:10.3389/fped.2021.753536)
Supplement: Supplementary file 2 [file Table_2.DOCX]

**Supplement 2**: Pediatric Patient-reported Experience Measures identified through grey literature

| **Country** | **Sources** | **Used PREMs** |
| --- | --- | --- |
| Australia | *Pediatric hospitals*   - Monash Children’s Hospital - Perth Children’s Hospital - Queensland Children’s Hospital - Royal Children’s Hospital Melbourne - Sydney Children’s Hospital - Women’s and Children’s Hospital | - MySay Healthcare Survey A statewide online survey for parents of public hospital patients (inpatient care or day-procedure). |
|  | *Health institute*   - Australian Government Department of Health |  |
| Austria | *Health institute*  Federal Ministry: Social Affairs, Health, Care and Consumer Practice | No information |
| Canada | *Pediatric hospitals*   - Alberta Children’s Hospital - British Colombia Children’s Hospital - Children’s Hospital of Eastern Ontario - Children’s Hospital of London Health Sciences Centre - CHU Sainte-Justine - Holland Bloorview Kids Rehabilitation Hospital - IWK Health Centre - Jim Pattison Children’s Hospital - McMaster Children’s Hospital - Montreal Children’s Hospital - Sickkids | - The Canadian Patient Experiences Survey on Inpatient Care   This bilingual PREM is based on the Hospital Consumer Assessment of Healthcare Providers and Systems (HCAHPS) survey. |
|  | *Health institutes*   - Alberta Health Services - Canadian Institute for Health Information - Provincial Health Services Authority |  |
| Israel | *Pediatric hospitals*   - Dana-Dwek Children’s Hospital Tel Aviv Sourasky Medical Center | No information |
| Japan | *Pediatric Hospitals*   - National Center for Child Health and Development | No information |
|  | *Health institute*   - Ministry of Health, Labour, Welfare |  |
| New Zealand | *Pediatric hospitals*   - Starship Hospital | No information |
|  | *Health institute*   - Ministry of Health |  |
| Sweden | *Pediatric hospitals*   - Astrid Lindgren Children’s Hospital - Huddinge Children’s Hospital - Queen Silvia Children’s Hospital - Sachsska Children’s Hospital - Skane University Hospital - Uppsala University Children’s Hospital | No information |
|  | *Health institute*   - Patient Centered Care Institute |  |
| The Netherlands | *Pediatric hospitals*   - Beatrix Children’s Hospital - Emma Children’s Hospital - Juliana’s Children’s Hospital - Princess Maxima Center for oncology - Sophia Children’s Hospital - VU Medical Center - University Hospital Maastricht - Wilhelmina Children’s Hospital - Willem-Alexander Children’s Hospital | - Patient Ervaringsmeting (PEM) This PREM is based on the Picker Institute surveys and is available for inpatient and outpatient visits. - Consumer Quality index (CQ-index) Different versions of the CQ-index are available for different disease groups. - BeterMeter   This survey is available for inpatient and outpatient visits. |
|  | *Health institute*   - Ministry of Health, Welfare and Sport |  |
| United Kingdom | *Pediatric hospitals*   - Alder Hey Children’s Hospital - Birmingham Children’s Hospital - Bristol Royal Hospital for Children - Derbyshire Children’s Hospital - Great Ormond Street Hospital - Evelina London Children’s Hospital - Noah’s Ark Children’s Hospital for Wales - Royal Aberdeen Children’s Hospital - Royal Alexandra Children’s Hospital - Royal Hospital for Sick Children - Royal Manchester Children’s Hospital - Saint Mary’s Hospital - Sheffield Children’s Hospital - Southampton Children’s Hospital - Tayside Children’s Hospital - West Glasgow Ambulatory Care Hospital | - National Patient Surveys   - Care Quality Commission Assessment All social care and healthcare have to be registered with the Care Quality Commission which assess hospitals on standards for patient care. - Healthcare Environment Inspectorate This questionnaire is distributed to patients, visitors and carers during inspection of the cleanliness of the hospital. - The under 16 Cancer Patient Experience Survey   This survey is developed in partnership with the Picker Institute Europe and address children and young people’s cancer experiences across England. |
|  | *Health institute*   - United Kingdom National Health Service (NHS) |  |
| United States of America | *Pediatric hospitals*   - Akron Children’s Hospital - Ann and Robert H. Lurie Children's Hospital - Arkansas Children’s Hospital - Boston Children’s Hospital - Bristol Myers Squibb Children’s Hospital - Children’s Hospital Colorado - Children’s Hospital Los Angeles - Children’s Hospital of Alabama - Children’s Hospital of Illionois - Children’s Hospital of Philadelphia - Children’s Hospital of Savannah at Memorial Health Medical Center - Children’s Mercy - Children’s National Medical Center - Cincinnati Children’s Hospital Medical Center - Cleveland Clinic Children’s Hospital Rehabilitation Center - Cohen Children’s Medical Center - Connecticut Children’s Medical Center - Cook Children’s Medical Center - Inova Children’s Hospital - Intermountain Primary Children’s Hospital - John Hopkins Children’s Center - Joseph M. Sanzari Children’s Hospital - Lucile Packard Children’s Hospital - Mattel Children’s Hospital UCLA - Miller Children’s & Women’s Hospital Long Beach - Monroe Carell Jr. Children’s Hospital - Morgan Stanley Children’s Hospital of New York-Presbyterian - Nationwide Children’s Hospital - Nicklaus Children’s Hospital - Peyton Manning Children’s Hospital - Phoenix Children’s Hospital - Rady Children’s Hospital - Riley Children’s Health - Seattle Children’s Hospital - St. Jude Children’s Research Hospital - St. Louis Children’s Hospital - Texas Children’s Hospital - The university of Chicago Comer Children’s Hospital - University of South Alabama Children’s & Women’s Hospital - UCSF Benioff Children’s Hospital - UPMC Children’s Hospital of Pitssburg - Valley Children’s Healthcare | - Child HCAHPS survey   Survey for parents/guardians about their experiences with pediatric inpatient care.   - Clinician and Group Consumer Assessment of Healthcare Providers and Systems (CG-CAHPS) survey   This survey is developed by the Agency of Healthcare Research and Quality and measures patient experiences with a range of health services.   - Press Ganey Satisfaction Survey   Hospitals are working together with Press Ganey Associaties, Inc., to measure patient experiences.   - National Research Corporation (NRC) Survey   Hospitals partnered with NRC Health, a third party patient experience company, to measure all aspects of patient experience.   - Picker surveys   Hospitals are working together with the Picker Institute to measure patient experiences.   - Pediatric Integrated Care Survey A survey to measure family experiences of care integration. This survey is used to improve the quality of care. - Adolescent Assessment of Preparation for Transition survey   This survey measures the quality of pre-transition counseling from adolescent-focused to adult-focused care for adolescents (16 and 17 years) with chronic conditions. |
|  | *Health Institutes*   - United Stated Department of Health and Human Services - National Institute of Health - Agency for Health Research and Quality |  |
